# Optimizing clinico-genomic disease prediction across ancestries: a machine learning strategy with Pareto improvement

**DOI:** 10.1186/s13073-024-01345-0

**Published:** 2024-06-04

**Authors:** Yan Gao, Yan Cui

**Affiliations:** 1 Department of Genetics, Genomics and Informatics, University of Tennessee Health Science Center, Memphis, TN 38163 USA; 2 Center for Integrative and Translational Genomics, University of Tennessee Health Science Center, Memphis, TN 38163 USA; 3 Center for Cancer Research, University of Tennessee Health Science Center, Memphis, TN 38163 USA

**Keywords:** Disease prediction, Transfer learning, Deep neural network, Health equity

## Abstract

**Background:**

Accurate prediction of an individual’s predisposition to diseases is vital for preventive medicine and early intervention. Various statistical and machine learning models have been developed for disease prediction using clinico-genomic data. However, the accuracy of clinico-genomic prediction of diseases may vary significantly across ancestry groups due to their unequal representation in clinical genomic datasets.

**Methods:**

We introduced a deep transfer learning approach to improve the performance of clinico-genomic prediction models for data-disadvantaged ancestry groups. We conducted machine learning experiments on multi-ancestral genomic datasets of lung cancer, prostate cancer, and Alzheimer’s disease, as well as on synthetic datasets with built-in data inequality and distribution shifts across ancestry groups.

**Results:**

Deep transfer learning significantly improved disease prediction accuracy for data-disadvantaged populations in our multi-ancestral machine learning experiments. In contrast, transfer learning based on linear frameworks did not achieve comparable improvements for these data-disadvantaged populations.

**Conclusions:**

This study shows that deep transfer learning can enhance fairness in multi-ancestral machine learning by improving prediction accuracy for data-disadvantaged populations without compromising prediction accuracy for other populations, thus providing a Pareto improvement towards equitable clinico-genomic prediction of diseases.

**Supplementary Information:**

The online version contains supplementary material available at 10.1186/s13073-024-01345-0.

## Background

Clinico-genomic prediction of diseases is essential to precision medicine. Traditionally, disease prediction was primarily based on epidemiological risk factors such as lifestyle variables and family history. Recent advances in high-throughput genotyping and genome sequencing technologies have enabled genome-wide association studies (GWAS) in large cohorts, providing the foundation for genomic disease prediction. However, more than 80% of the existing GWAS data were acquired from individuals of European descent [1–7], and the ancestral (or ethnic) diversity in GWAS has not improved in recent years [1, 5]. The lack of adequate genomic data for non-European populations, who make up approximately 84% of the world’s population, results in low-quality artificial intelligence (AI) models for these data-disadvantaged populations (DDPs). Genomic data inequality is thus emerging as a significant health risk and a new source of health disparities [7, 8].

Genomic prediction models based on GWAS data from predominantly European ancestry have limited applicability to other ancestry groups [5, 9–15]. Recent studies indicate that cross-ancestry generalizability in polygenic models can be improved by calibrating parameters for genetic effect sizes or model sparsity (or shrinkage) patterns across ancestry groups [16–24]. However, the limitations of these models, including assumptions of linearity, additivity, and distribution normality, restrict their ability to learn and transfer complex representations across different ancestry groups. In contrast, deep neural networks have much higher model capacities to capture complex, non-linear relationships and are more adept at transfer learning [25].

We have developed a framework to address the impact of biomedical data inequality and distribution shift on multi-ancestral machine learning and demonstrated its effectiveness in cancer progression and survival prediction tasks [7, 8, 26]. This study extends that framework to optimize clinico-genomic disease prediction across ancestries. We compare the performance of different models within and across the multi-ancestral machine learning schemes and find that deep transfer learning can significantly improve disease prediction accuracy for data-disadvantaged ancestry groups without compromising the prediction accuracy for other ancestry groups. Therefore, our study shows that deep transfer learning may provide a Pareto improvement [27] for optimizing multi-ancestral genomic prediction of diseases.

## Methods

### Clinico-genomic datasets for multi-ancestral machine learning experiments

The genotype, phenotype, and clinical data for lung cancer, prostate cancer, and Alzheimer’s disease were retrieved from the dbGaP datasets: OncoArray Consortium—Lung Cancer Studies (phs001273.v3.p2) [28–32], OncoArray: Prostate Cancer (phs001391.v1.p1) [31], Alzheimer’s Disease Genetics Consortium Genome Wide Association Study -NIA Alzheimer’s Disease Centers Cohort (phs000372.v1.p1) [33, 34], and Columbia University Study of Caribbean Hispanics with Familial and Sporadic Late Onset Alzheimer’s disease (phs000496.v1.p1) [35–37]. Genetic ancestries of the GWAS participants determined using GRAF-pop [38] were also retrieved from the dbGaP. We implemented a standard quality-control process [39] and used the PLINK software [40] to identify disease-associated single nucleotide polymorphisms (SNPs). We excluded SNPs with a missing rate > 20%, a minor allele frequency (MAF) < 0.05, or a Hardy–Weinberg Equilibrium (HWE) *p*-value < 10^−5^. We also removed the samples with sex discrepancy or a missing SNP rate > 20%. To reduce data redundancy from linkage disequilibrium (LD), we used a sliding window of 50 SNPs, with a step length of 5 SNPs and an LD cut-off coefficient of 0.2. The LD pruning procedure involved examining each window for pairs of variants with squared correlation exceeding a predefined threshold. Pairs meeting this criterion were identified, and a greedy algorithm was applied to prune variants from the window until no pairs with squared correlation above the threshold remained. For a pair of SNPs in high LD, we retained the SNP with the lower *p*-value and removed the other. The resulting datasets for the machine learning experiments are summarized in Table 1.

**Table 1:** Multi-ancestral datasets for clinico-genomic prediction of diseases

**Lung cancer (dataset 1)**		
European	Case	9675
	Control	8107
	Age range	15–90 +
	Male	11,605
	Female	6177
East Asian	Case	1363
	Control	801
	Age range	2–90 +
	Male	1924
	Female	240
**Prostate cancer (dataset 2)**		
European	Case	28,417
	Control	19,796
	Age range	19.0–93.0
	Male	48,213
	Female	0
African American	Case	1231
	Control	2017
	Age range	21.0–89.0
	Male	3248
	Female	0
**Alzheimer’s disease (dataset 3)**		
European	Case	3493
	Control	1518
	Male	2211
	Female	2800
Latin American	Case	1096
	Control	1222
	Male	789
	Female	1529
**Alzheimer’s disease (dataset 4)**		
European	Case	3493
	Control	1518
	Male	2211
	Female	2800
African American	Case	682
	Control	821
	Male	442
	Female	1061

We assembled four machine learning datasets for multi-ancestral clinico-genomic prediction of diseases: a lung cancer dataset with European and East Asian populations, a prostate cancer dataset with European and African American populations, and two Alzheimer’s disease datasets (one with European and Latin American populations, another with European and African American populations). Each dataset comprised two subpopulations: the European (EUR) population and a data-disadvantaged population (DDP).

We split each dataset into three parts: the training set, the validation set, and the testing set, each comprised of 80%, 10%, and 10% of the individuals, respectively, stratified by ancestry and class label (case/control). The training set was used to learn the underlying patterns in the data by fitting the machine learning model’s parameters. The validation set was used to tune the hyperparameters during model training to prevent overfitting. The testing set was used to assess the performance of the model after the training process was complete.

### Feature selection

We conducted the association analysis on the training set using the additive logistic regression model provided by the PLINK software. The feature mask, ANOVA *F*-value, and *p*-values were generated using the SelectKBest function from the scikit-learn machine learning software library in Python. The feature set for lung cancer comprises the top 500 (or 1000) SNPs identified in the association analysis and the clinical variables of age, sex, and smoking status. Similarly, the feature set for prostate cancer includes the top 500 (or 1000) SNPs from the association analysis and the clinical variables of age and family history.

We retrieved the lists of SNPs of 16 polygenic scores for Alzheimer’s disease (AD) from the Polygenic Score Catalog [41] (with accession numbers PGS000025, PGS000026, PGS000334, PGS000779, PGS000811, PGS000812, PGS000823, PGS000876, PGS000898, PGS000945, PGS001348, PGS001349, PGS001775, PGS001828, PGS002280, and PGS002731), which were published in recent studies [13, 42–53]. To prevent information leakage in feature selection [54, 55], polygenic scores derived from the dbGaP datasets used in this study were excluded. We compiled a list of 22 SNPs present in any of the 16 polygenic scores and the dbGaP datasets for Alzheimer’s disease used in this study. The AD feature set for our machine learning experiments includes the 22 SNPs, sex, and allele value for the Apolipoprotein E (APOE) gene. The APOE gene is crucial in AD risk and progression, primarily influencing amyloid-beta plaque accumulation, tau pathology, and lipid metabolism in the brain [56]. It has three common isoforms encoded by the ε2 (associated with reduced AD risk), ε3 (neutral), and ε4 (associated with increased AD risk) alleles.

### Synthetic data

We generated synthetic datasets with built-in data inequality and distribution shifts across ancestry groups. Each synthetic dataset (*D*) contains data from two ancestry populations:$D={D}_{1} \bigcup {D}_{2}$,${D}_{1}={\{{x}_{ij}, {y}_{i}\}}_{i=1}^{n}$,${D}_{2}={\{{x}_{ij}^{\prime}, {y}_{i}^{\prime}\}}_{i=1}^{{n}^{\prime}}$. Here, ${D}_{1}$ represents the EUR population, ${D}_{2}$ represents a data-disadvantaged population (DDP), $n$ and ${n}^{\prime}$ are the numbers of individuals in the EUR and DDP respectively, ${x}_{ij}$ is the ${j}^{th}$ feature of individual $i$ in the European population, ${x}_{ij}^{\prime}$ is the ${j}^{th}$ feature of individual $i$ in the DDP, ${y}_{i}$ is the case/control status of individual $i$ in the European population, ${y}_{i}^{\prime}$ is the case/control status of individual $i$ in the DDP. The case/control status of the ${i}^{th}$ EUR individual was generated using $${y}_{i}=\left\{\begin{array}{c}1\ \text{if}\ {g}_{i}>\text{thr}\\ 0\ \text{otherwise}\end{array}\right.$$, where${g}_{i}=h{\sum }_{j=1}^{m}{w}_{j}*{x}_{ij}+ a\sqrt{1-{h}^{2}}* \zeta$*,* $thr$ is the parameter to determine case-to-control ratio, ${w}_{j}$ is the effect size of the ${j}^{th}$ SNP, $\zeta$ was sampled from a standard normal distribution over [-1,1], $a$ is a scaling factor for variance normalization, $m$ is the number of SNPs, and ${h}^{2}$ is the heritability. Similarly, the label of the ${i}^{th}$ DDP individual was generated using the function $${y}_{i}^{\prime}=\left\{\begin{array}{c}1\ \text{if}\ {g}_{i}^{\prime}>{\text{thr}^{\prime}} \\ 0\ \text{otherwise}\end{array}\right.$$, where${g}_{i}^{\prime}=h{\sum }_{j=1}^{m}{w}_{j}^{\prime}*{x}_{ij}^{\prime} +{a}^{\prime}\sqrt{1-{h}^{2}}* \zeta$, ${\text{thr}^{\prime}}$ is the parameter to determine case-to-control ratio, ${w}_{j}^{\prime}$ is the effect size of the *j*th SNP, ${a}^{\prime}$ is a scaling factor for variance normalization. The genetic effect vector $W=\left[{w}_{1},{w}_{2},{w}_{3},\dots {w}_{m}\right]$ for EUR was randomly sampled from a normal distribution over$[-\text{1,1}]$. ${W}^{\prime}=\left[{w}_{1}^{\prime},{w}_{2}^{\prime},{w}_{3}^{\prime},\dots {w}_{m}^{\prime}\right]$ for the DDP was generated using${W}^{\prime}=\rho *W+\sqrt{1-{\rho }^{2}}* {\zeta }^{\prime}$, where $\rho$ is the correlation of the genetic effect sizes between EUR and the DDP, ${\zeta }^{\prime}$ was randomly sampled from a standard normal distribution over [-1,1].

The feature matrices were assembled using the simulated genotype data from the Harvard Dataverse [24, 57]. The dataset includes five ancestry populations: African (AFR), Admixed American (AMR), East Asian (EAS), European (EUR), and South Asian (SAS), each consisting of 120,000 individuals. After applying a quality control process to remove SNPs with MAF < 0.05 or HWE *p*-value < 10^−4^, we randomly selected 500 SNPs as features, and 10,000 EUR individuals and 2000 individuals from each of the four non-EUR populations to construct the synthetic datasets. We calculated the case/control status for various heritability (${h}^{2}=0.5\ \text{and}\ {h}^{2}$ = 0.25) and genetic correlation ($\rho$) values. The parameter $\rho$ is determined using a function of genetic distance. The genetic distance between EUR and the *k*th DDP is ${d}_{k} ={\sum }_{j=1}^{500}|{F}_{j}^{\text{EUR}}-{F}_{j}^{{\text{DDP}}_{k}}|$, where ${F}_{j}^{\text{EUR}}$ is the frequency of the minor allele of *j*th SNP in the EUR population, and ${F}_{j}^{{\text{DDP}}_{k}}$ is the frequency of the same allele in the *k*th DDP. The genetic distances were calculated as ${d}_{0}=$ 42.7 for EUR and AMR, ${d}_{1}=$ 46.1 for EUR and SAS, ${d}_{2}=$ 80.6 for EUR and EAS, and ${d}_{3}=$ 94.1 for EUR and AFR. The function ${\rho }_{k}= {\rho }_{0}{\left(\frac{{d}_{0}}{{d}_{k}}\right)}^{r}$ was used to generate the $\rho$ values, with ${\rho }_{0}=0.8$. We set $r=0.5$ to generate the $\rho$ values for synthetic datasets SD1-SD8 and SD1*-SD8*, and $r=1.0$ to generate the $\rho$ values for synthetic datasets SD9-SD16 and SD9*-SD16*. The case-to-control ratio is 1:1 for synthetic datasets SD1-SD16, and 1:4 for synthetic datasets SD1*-SD16*, respectively.

#### Multi-ancestral machine learning schemes and experiments

A multi-ancestral machine learning strategy should be optimized for both prediction accuracy and equity, encompassing disparity detection and mitigation. The primary challenge lies in handling datasets that contain multiple ancestry groups with data inequality and distribution shifts. Therefore, how to utilize data from different ancestry groups is crucial in a multi-ancestral machine learning strategy. We have categorized multi-ancestral (or multi-ethnic) machine learning schemes based on the way they utilize data from different subpopulations [7, 8] (Table 2). Mixture learning indistinctly uses data from all ancestral populations for model training and testing. Independent learning trains and tests a model for each ancestral group separately. In naïve transfer, the model trained on source domain (EUR) data was applied directly to the target domain (DDP) without adaptation. In transfer learning, a model is first trained on the data of the European population (source domain), then the knowledge (or representation) learned from the source domain is transferred to facilitate model development for a DDP (target domain). In this work, we assessed the performance of these multi-ancestral machine learning schemes in the context of clinico-genomic disease prediction. Our experiments are designed to address both bias detection and mitigation: experiments with mixture and independent learning schemes aim to detect model performance disparities, while those involving transfer learning focus on disparity mitigation.

**Table 2:** Multi-ancestral machine learning schemes and experiments

Multi-ancestral machine learning scheme	Experiment	Ancestral composition	
		**Training data**	**Testing data**
Mixture learning	Mix0	EUR + DDP	EUR + DDP
	Mix1		EUR
	Mix2		DDP
Independent learning	Ind1	EUR	EUR
	Ind2	DDP	DDP
Naive transfer	NT	EUR	DDP
Transfer learning	TL	EUR (source domain) DDP (target domain)	DDP

*EUR *European, *DDP *Data-disadvantaged population

### Deep neural network (DNN) and DNN-based transfer learning

We constructed the deep neural network (DNN) models using the Keras (https://keras.io/) and Tensorflow (https://www.tensorflow.org/) software libraries. The DNN models were designed with a pyramid architecture [58] consisting of four layers: an input layer with K nodes for the input features of genetic and other risk factors, two hidden layers, including a fully connected layer with 100 nodes followed by a dropout layer [59], and a logistic regression output layer. We used the stochastic gradient descent (SGD) algorithm with a learning rate of 0.25 to minimize a loss function consisting of a binary cross-entropy term and two regularization terms: $l\left(W\right)= -\sum ({y}_{i} log\left({\widehat{y}}_{i}\right)+\left(1-{y}_{i} \right)\text{ log}(1-{\widehat{y}}_{i}))+ {\lambda }_{1}\left|W\right| + {\lambda }_{2}{\Vert W\Vert }_{2}$, where ${y}_{i}$ is the observed control/case status for individual $i$, ${\widehat{y}}_{i}$ is the predicted control/case status for individual $i$, and $W$ represents the weights in the DNN model. We applied the ReLU activation function $f(x) = \text{max}(0, x)$ to the hidden layer output to avoid the vanishing gradient problem. For each dropout layer, we set the dropout probability $p=0.5$ to randomly omit half of the weights during the training to reduce the collinearity between feature detectors. We split the data into multiple mini-batches (batch size = 32) for training to speed up the computation and improve the model prediction performance. We set the maximum number of iterations at 200 and applied the Nesterov momentum [60] method (with momentum = 0.9) to prevent premature stopping. We set the learning rate decay factor at 0.003. We also used early stopping with a patience value of 200 iterations to monitor the validation accuracy during model fitting. The two regularization terms ${\lambda }_{1}$ and ${\lambda }_{2}$ were set at 0.001.

In transfer learning, knowledge and representation learned from the source domain are transferred to assist the learning task for the target domain [61–68]. In each task, we used the EUR as the source domain and the DDP as the target domain. We used a supervised fine-tuning algorithm for transfer learning. We first pretrained a DNN model using the source domain data: $M \sim f({Y}_{\text{Source}}|{X}_{\text{Source}})$, where $M$ represents the pretrained model, ${X}_{\text{Source}}$ and ${Y}_{\text{Source}}$ represent the features and the class labels in the source domain, respectively. We trained the DNN model using the parameters described above. After the pretraining, we fine-tuned the model with the backpropagation method using the target domain data: ${M}^{\prime}=\text{fine}\_\text{tuning}\left(M \right| {Y}_{\text{Target}},{X}_{\text{Target}})$, where ${M}^{\prime}$ represents the final model, ${X}_{\text{Target}}$ and ${Y}_{\text{Target}}$ represent the features and the class labels in the target domain, respectively.

### Logistic regression (LR) and LR-based transfer learning

Logistic regression models, capable of incorporating genetic and clinical factors, have been widely used in the clinico-genomic prediction of binary disease outcomes [69]. We used the logistic regression model with L2 regularization from the Python scikit-learn library [70]. For LR-based transfer learning, we adapted the TL_PRS [21] model, a linear polygenic model pretrained on EUR genomic data and fine-tuned on data from other ancestry groups to improve cross-population transferability. The adapted model, integrating additional terms for clinical variables, can be expressed as: ${\widehat{Y}}_{i}={\sum }_{j=1}^{m}{G}_{ij}{\beta }_{j}+{\sum }_{k=1}^{M}{C}_{ij}{\gamma }_{k}+ \epsilon ={\sum }_{j=1}^{m}{G}_{ij}({\beta }_{j}^{\text{pre}}+{\tau }_{j})+{\sum }_{k=1}^{M}{C}_{ij}({\gamma }_{k}^{\text{pre}}+{\delta }_{k})+ \epsilon$, where ${\widehat{Y}}_{i}$ is the predicted phenotype of the *i*th sample in the target ancestry group, $m$ is the number of SNPs, ${G}_{ij}$ is the genotype of the *j*th SNP of individual $i$, ${\beta }_{j}$ is the effect size of the *j*th SNP in the target population (a DDP), $M$ is the number of clinical variables, ${C}_{ik}$ is the *k*th clinical variable of individual $i$, ${\gamma }_{k}$ is the effect size of the *k*th clinical variable in the target population, $\epsilon$ is the white noise from a standard normal distribution, ${\beta }_{j}^{\text{pre}}$ refers to the estimated effect size of the *j*th SNP in EUR, ${\tau }_{j}$ is the difference between ${\beta }_{j}^{\text{pre}}$ and ${\beta }_{j}$, ${\gamma }_{k}^{\text{pre}}$ refers to the estimated effect size of the *k*th clinical variable in EUR, and ${\delta }_{k}$ is the difference between ${\gamma }_{k}^{\text{pre}}$ and ${\gamma }_{k}$. For training the LR-based models, we utilized the stochastic average gradient solver which can provide efficient convergence for large datasets [71].

### Application of PRS-CSx

PRS-CSx (Polygenic Risk Score with Cross-Study summary statistics) is a recently developed method that utilizes summary statistics from genome-wide association studies (GWAS) across multiple populations to construct a robust and generalizable polygenic score [16]. We evaluated the performance of PRS-CSx using our synthetic datasets. In our experiments, we followed the guidelines provided in the PRS-CSx GitHub repository (https://github.com/getian107/PRScsx). We used the software package Plink (version 1.9) to generate GWAS summary statistics for the two ancestry groups in each dataset. Subsequently, the GWAS summary statistics, combined with the LD references, were used as input for the PRS-CSx Python scripts to perform polygenic prediction.

### Evaluation and comparison of machine learning model performance

We used five metrics to assess and compare the performance of machine learning models: the area under the receiver operating characteristic curve (AUROC), the area under the precision-recall curve (AUPR), Tjur’s *R*^2^, positive predictive value (PPV), and negative predictive value (NPV). AUROC and AUPR are global metrics for assessing the performance of predictive models, summarizing the model’s performance across all possible classification thresholds. Tjur’s *R*^2^, also known as the coefficient of discrimination, quantifies a model’s ability to distinguish between binary outcomes [72]. Compared to other pseudo-R^2^, this metric provides a more direct measure of the model’s ability to differentiate between binary outcomes and is asymptotically equivalent to the traditional *R*^2^ measures at large sample sizes [72]. PPV and NPV are threshold-dependent metrics that assess model performance at a single, specific classification threshold. We used the Youden’s Index [73] to determine the optimal classification threshold for calculating PPV and NPV. The Youden’s Index is defined as $J=\text{sensitivity}+\text{specificity}-1$. The threshold that maximizes Youden’s Index is considered optimal as it maximizes the overall correct classification rate while balancing sensitivity (true positive rate) and specificity (true negative rate). All these performance metrics have been used in recent studies to evaluate multi-ancestral clinico-genomic prediction of diseases [50, 74, 75].

The evaluation of multi-ancestral machine learning models often involves comparing model performance across ancestry groups with different disease prevalences. AUROC, which is independent of prevalence (or class distribution), is therefore used as the primary metric for assessing model performance in this study. While other metrics may vary in their sensitivity to prevalence, incorporating multiple metrics provides a more comprehensive view of model performance.

We conducted 20 independent runs for each experiment, calculated the mean values of the metrics of the 20 runs, and used a one-sided Wilcoxon rank sum test to calculate *p*-values to assess the statistical significance of the performance differences between the various experiments. Additionally, the one-sided Wilcoxon signed-rank test was used to evaluate the performance differences across multiple matched experiments and/or datasets.

## Results

### Multi-ancestral clinico-genomic prediction of diseases

**Fig. 1:**
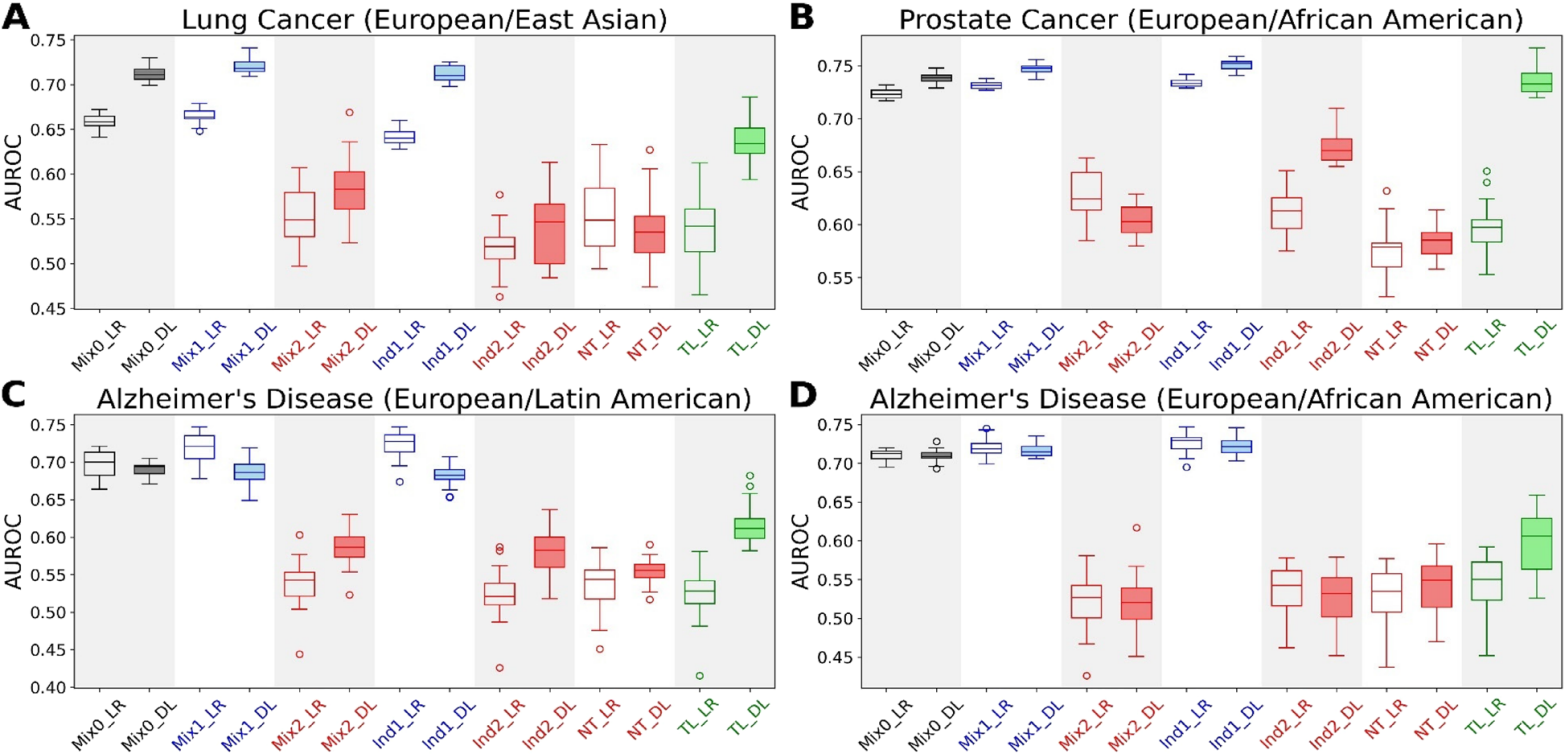
Multi-ancestral clinico-genomic prediction of **A** lung cancer involving European and East Asian populations, **B** prostate cancer involving European and African American populations, **C** Alzheimer’s disease involving European and Latin American populations, and **D** Alzheimer’s disease involving European and African American populations. Each box plot represents the machine learning model performance (AUROC) of 20 independent runs. LR, logistic regression; DL, deep learning. Mix0, Mix1, Mix2, Ind1, Ind2, NT, and TL are the machine learning experiments outlined in Table 2

We assembled four datasets for the multi-ancestral clinico-genomic prediction of lung cancer, prostate cancer, and Alzheimer’s disease, utilizing data from dbGaP (Table 1). These datasets were used in machine learning tasks to predict disease status (case/control). The specific multi-ancestral machine learning schemes and experiments are outlined in Table 2. In each experiment, we applied two machine learning models: a logistic regression (LR)-based model and a deep learning (DL)-based model (Table 3, Fig. 1). In both LR- and DL-based experiments, the mixture and independent learning schemes resulted in significant model performance disparity gaps between EUR and DDPs (Table 4). The performance disparity gap is defined as ${G=\overline{\text{AUROC}} }_{\text{EUR}}-{\overline{\text{AUROC}} }_{\text{DDP}}$, where ${\overline{\text{AUROC}} }_{\text{EUR}}$ and ${\overline{\text{AUROC}} }_{\text{DDP}}$ are the mean AUROC for the EUR and DDP in an experiment, respectively.

**Table 3:** Multi-ancestral clinico-genomic prediction of diseases

Datasets		Area under ROC curve (LR, DL)						
**Disease**	**DDP**	**Mix0**	**Mix1**	**Mix2**	**Ind1**	**Ind2**	**NT**	**TL**
Lung cancer	East Asian	0.66, 0.71	0.66, 0.72	0.55, 0.58	0.64, 0.71	0.52, 0.54	0.55, 0.54	0.54, 0.64
Prostate cancer	African American	0.72, 0.74	0.73, 0.75	0.63, 0.60	0.73, 0.75	0.61, 0.67	0.58, 0.58	0.60, 0.74
Alzheimer’s disease	Latin American	0.70, 0.69	0.72, 0.69	0.54, 0.58	0.72, 0.68	0.52, 0.58	0.53, 0.55	0.53, 0.62
Alzheimer’s disease	African American	0.71, 0.71	0.72, 0.72	0.52, 0.52	0.73, 0.72	0.54, 0.53	0.53, 0.54	0.54, 0.60

*LR* Logistic regression, *DL* Deep learning, *DDP* Data-disadvantaged population. Mix0, Mix1, Mix2, Ind1, Ind2, NT, and TL are the machine learning experiments outlined in Table 2

**Table 4:** Disparity detection and mitigation in multi-ancestral clinico-genomic prediction

Key observations	Comparison	Mean difference in AUROC			
		**LC**	**PC**	**AD1**	**AD2**
Performance disparity gap between EUR and DDPs in mixture and independent learning	Mix1_LR vs Mix2_LR	**0.11**	**0.10**	**0.18**	**0.20**
	Mix1_DL vs Mix2_DL	**0.14**	**0.14**	**0.10**	**0.20**
	Ind1_LR vs Ind2_LR	**0.13**	**0.12**	**0.20**	**0.19**
	Ind1_DL vs Ind2_DL	**0.17**	**0.08**	**0.10**	**0.20**
Improvement from DL-based transfer learning	TL_DL vs Mix2_DL	**0.06**	**0.13**	**0.03**	**0.08**
	TL_DL vs Ind2_DL	**0.10**	**0.06**	**0.03**	**0.07**
	TL_DL vs NT_DL	**0.10**	**0.15**	**0.06**	**0.06**
Improvement from LR-based transfer learning	TL_LR vs Mix2_LR	-0.02	-0.03	-0.01	**0.02**
	TL_LR vs Ind2_LR	**0.02**	-0.02	0.00	0.01
	TL_LR vs NT_LR	-0.01	**0.02**	-0.01	0.02
Performance difference between DL- and LR-based transfer learning	TL_DL vs TL_LR	**0.10**	**0.14**	**0.09**	**0.06**

The statistically significant (*p*-value less than 0.05) performance differences are highlighted using bold font. LC, PC, AD1, and AD2 are the clinico-genomic datasets; *LC* Lung cancer (European and East Asian populations), *PC* Prostate cancer (European and African American populations), *AD1* Alzheimer’s disease (European and Latin American populations), *AD2* Alzheimer’s disease (European and African American populations), *LR* Logistic regression, *DL* Deep learning; Mix0, Mix1, Mix2, Ind1, Ind2, NT, and TL are the machine learning experiments outlined in Table 2

In mixture learning, the performance disparity gaps from the LR (Mix1_LR vs Mix2_LR) and DL (Mix1_DL vs Mix2_DL) models and the *p*-values for the statistical significance of the performance disparities are:

- Lung cancer (European and East Asian populations): ${G}_{\text{Mix}\_\text{LR}}=0.11$ ($p={3.36 \times 10}^{-8}$) and ${G}_{\text{Mix}\_\text{DL}}=0.14$ ($p={3.36 \times 10}^{-8}$)
- Prostate cancer (European and African American populations): ${G}_{\text{Mix}\_\text{LR}}=0.10$ ($p={3.32 \times 10}^{-8}$) and ${G}_{\text{Mix}\_\text{DL}}=0.14$ ($p={3.34 \times 10}^{-8}$)
- Alzheimer’s disease (European and Latin American populations): ${G}_{\text{Mix}\_\text{LR}}=0.18$ ($p={3.38\times10}^{-8}$) and ${G}_{\text{Mix}\_\text{DL}}=0.10$ ($p={3.35 \times 10}^{-8}$)
- Alzheimer’s disease (European and African American populations): ${G}_{\text{Mix}\_\text{LR}}=0.20$ ($p={3..39\times10}^{-8}$) and ${G}_{\text{Mix}\_\text{DL}}=0.20$ ($p={3.32 \times 10}^{-8}$)

In independent learning, the performance disparity gaps (Ind1_LR vs Ind2_LR, and Ind1_DL vs Ind2_DL) and *p*-values are:

- Lung cancer (European and East Asian populations): ${G}_{\text{Ind}\_\text{LR}}=0.13$ ($p={3.37 \times 10}^{-8}$) and ${G}_{\text{Ind}\_\text{DL}}=0.17$ ($p={3.34 \times 10}^{-8}$)
- Prostate cancer (European and African American populations): ${G}_{\text{Ind}\_\text{LR}}=0.12$ ($p={3.34 \times 10}^{-8}$) and ${G}_{\text{Ind}\_\text{DL}}=0.08$ ($p={3.32 \times 10}^{-8}$)
- Alzheimer’s disease (European and Latin American populations): ${G}_{\text{Ind}\_\text{LR}}=0.20$ ($p={3.36 \times 10}^{-8}$) and ${G}_{\text{Ind}\_\text{DL}}=0.10$ ($p={3.36 \times 10}^{-8}$)
- Alzheimer’s disease (European and African American populations): ${G}_{\text{Ind}\_\text{LR}}=0.19$ ($p={3.39 \times 10}^{-8}$) and ${G}_{\text{Ind}\_\text{DL}}=0.20$ ($p={3.34 \times 10}^{-8}$)

The naïve transfer approach, in which the model trained on source domain (EUR) data was applied directly to the target domain (DDP) without adaptation, also resulted in low performance for the DDPs. This is consistent with previous findings demonstrating the limited generalizability of models trained on EUR data to other ancestry groups [5, 9–15].

Using performance of mixture learning, independent learning, and naïve transfer for the DDP (Mix2, Ind2, and NT) as baselines, we quantified the improvement in model performance by DL- and LR-based transfer learning:

- ${{I}_{\text{Mix}\_\text{DL}}=\overline{\text{AUROC}} }_{\text{TL}\_\text{DL}}-{\overline{\text{AUROC}} }_{\text{Mix}2\_\text{DL}}$ is the performance improvement over Mix2 (TL_DL vs Mix2_DL).
- ${{I}_{\text{Ind}\_\text{DL}}=\overline{\text{AUROC}} }_{\text{TL}\_\text{DL}}-{\overline{\text{AUROC}} }_{\text{Ind}2\_\text{DL}}$ is the performance improvement over Ind2 (TL_DL vs Ind2_DL).
- ${{I}_{\text{NT}\_\text{DL}}=\overline{\text{AUROC}} }_{\text{TL}\_\text{DL}}-{\overline{\text{AUROC}} }_{\text{NT}\_\text{DL}}$ is the performance improvement over NT (TL_DL vs NT_DL).
- ${{I}_{\text{Mix}\_\text{LR}}=\overline{\text{AUROC}} }_{\text{TL}\_\text{LR}}-{\overline{\text{AUROC}} }_{\text{Mix}2\_\text{LR}}$ is the performance improvement over Mix2 (TL_LR vs Mix2_ LR).
- ${{I}_{\text{Ind}\_\text{LR}}=\overline{\text{AUROC}} }_{\text{TL}\_\text{LR}}-{\overline{\text{AUROC}} }_{\text{Ind}2\_\text{LR}}$ is the performance improvement over Ind2 (TL_ LR vs Ind2_ LR).
- ${{I}_{\text{NT}\_\text{LR}}=\overline{\text{AUROC}} }_{\text{TL}\_\text{LR}}-{\overline{\text{AUROC}} }_{\text{NT}\_\text{LR}}$ is the performance improvement over NT (TL_ LR vs NT_ LR).

The improvements by DL-based transfer learning and the corresponding *p*-values are:

- Lung cancer (European and East Asian populations): ${I}_{\text{Mix}}=0.06$ ($p={1.02 \times 10}^{-5}$), ${I}_{\text{Ind}}=0.10$ ($p={4.55 \times 10}^{-8}$), and ${I}_{\text{NT}}=0.10$ ($p={1.28 \times 10}^{-7}$)
- Prostate cancer (European and African American populations): ${I}_{\text{Mix}}=0.13$ ($p={3.37 \times 10}^{-8}$), ${I}_{\text{Ind}}=0.06$ ($p={3.37 \times 10}^{-8}$), and ${I}_{\text{NT}}=0.15$ ($p={3.38 \times 10}^{-8}$)
- Alzheimer’s disease (European and Latin American populations): ${I}_{\text{Mix}}=0.03$ ($p={2.66 \times 10}^{-4}$), ${I}_{\text{Ind}}=0.03$ ($p={4.81 \times 10}^{-4}$), and ${I}_{\text{NT}}=0.06$ ($p={4.57 \times 10}^{-8}$)
- Alzheimer’s disease (European and African American populations): ${I}_{\text{Mix}}=0.08$ ($p={4.29 \times 10}^{-6}$), ${I}_{\text{Ind}}=0.07$ ($p={7.49 \times 10}^{-6}$), and ${I}_{\text{NT}}=0.06$ ($p={1.87 \times 10}^{-4}$)

While the DL-based transfer learning significantly improved the model performance for the DDPs, the improvements from LR-based transfer learning are largely insignificant, even with negative ${I}_{\text{Mix}\_\text{LR}}$, ${I}_{\text{Ind}\_\text{LR}}$, and ${I}_{\text{NT}\_\text{LR}}$ in many cases (Table 4).

The performance difference between DL-based and LR-based transfer learning is ${D=\overline{\text{AUROC}} }_{\text{TL}\_\text{DL}}-{\overline{\text{AUROC}} }_{\text{TL}\_\text{LR}}$ The performance differences between DL-based and LR-based transfer learning (TL_DL vs TL_LR) and the *p*-values are:

- Lung cancer (European and East Asian populations): $D=0.10$ ($p={5.31 \times 10}^{-8}$)
- Prostate cancer (European and African American populations): $D=0.14$ ($p={3.38 \times 10}^{-8}$)
- Alzheimer’s disease (European and Latin American populations): $D=0.09$ ($p={3.93 \times 10}^{-8}$)
- Alzheimer’s disease (European and African American populations): $D=0.06$ ($p={2.95 \times 10}^{-4}$)

In summary, the key observations from our experiments are (1) the mixture and independent learning schemes led to significant model performance disparity gaps between EUR and DDPs, regardless of the specific machine learning models (LR-based or DL-based); (2) DL-based transfer learning significantly improved model performance for the DDPs; (3) LR-based transfer learning did not achieve such substantial improvements; and (4) DL-based transfer learning significantly outperformed LR-based transfer learning (Table 4).

Four additional performance metrics, AUPR, Tjur’s *R*^2^, PPV, and NPV, were used to confirm these key observations. In the model performance assessment and comparison using AUPR and Tjur’s *R*^2^ (Table S1, Table S2, and Fig. S1), all the key observations are consistent with those obtained using AUROC as the performance metric, except in the case of lung cancer dataset, where the logistic regression (LR)-based model did not exhibit significant performance disparity gaps (measured using AUPR) in mixture and independent learning (Table S3).

PPV and NPV are dependent on disease prevalence. The sensitivity and specificity metrics can be used, along with disease prevalence in a population of interest, to calculate adjusted PPV and NPV [74]:

$$\text{PPV}=\frac{\text{sensitivity}\times \text{prevalence}}{\text{sensitivity}\times \text{prevalence}+(1-\text{specificity})\times (1-\text{prevalence})}$$

$$\text{NPV}= \frac{\text{specificity}\times (1-\text{prevalence})}{\text{specificity}\times (1-\text{prevalence})+(1-\text{sensitivity})\times \text{prevalence}}$$

This adjustment accounts for variations in disease prevalence across different populations, ensuring that the predictive values are more precisely aligned with the specific context. Prevalence is often reported for chronic diseases such as Alzheimer’s disease but not commonly used for lung and prostate cancers. We calculated sensitivity and specificity (Table S4 and S5) and used these metrics along with ancestry-specific prevalence to compute adjusted PPV and NPV for Alzheimer’s disease across all the experiments except Mix0 where the target population comprises individuals from two different ancestry groups (Table S6 and S7). The prevalence of Alzheimer’s disease among individuals aged 65 and older in the USA was used: African American (13.8%), Latino (12.2%), and European (10.3%) [76]. All the key observations derived from the prevalence-adjusted PPV and NPV are consistent with those obtained using AUROC (Table S8).

Additionally, we conducted the same sets of experiments on the lung cancer and prostate cancer datasets that include the top 1000 SNPs and found that the key observations remained consistent when using more SNP features (Fig. S2 and Fig. S3).

### Multi-ancestral machine learning experiments on synthetic data

**Fig. 2:**
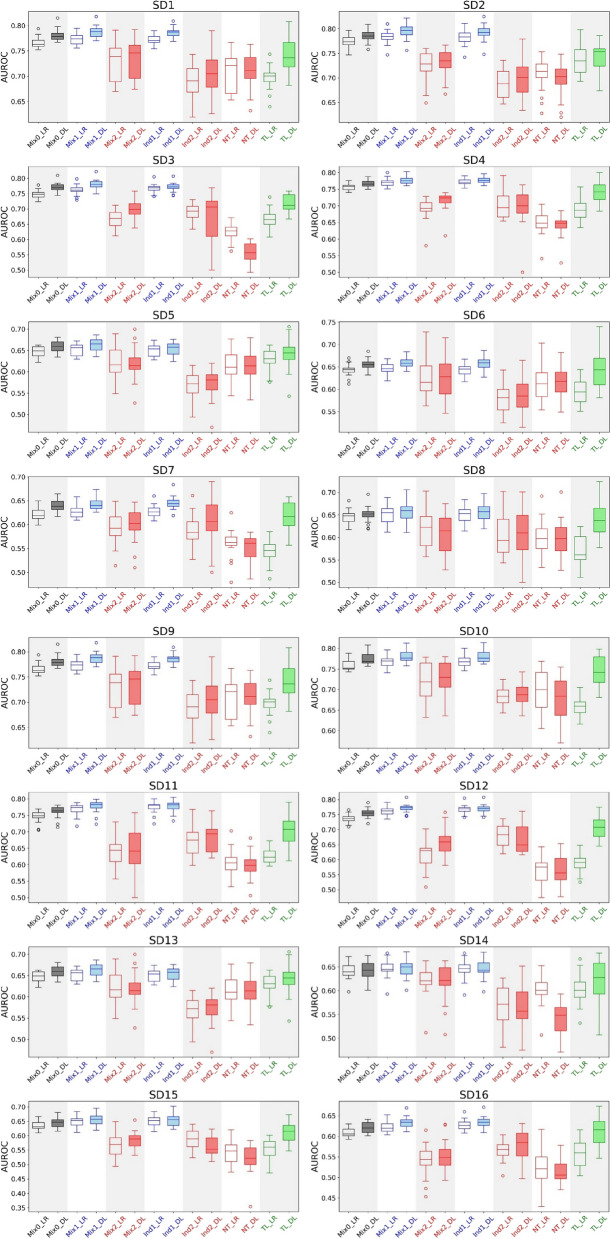
Multi-ancestral machine learning experiments on synthetic dataset compendium SD. Each box plot represents the machine learning model performance (AUROC) of 20 independent runs. LR, logistic regression; DL, deep learning. Mix0, Mix1, Mix2, Ind1, Ind2, NT, and TL are the machine learning experiments outlined in Table 2

To test the generalizability of these observations across diverse conditions, we conducted the same multi-ancestral machine learning experiments (as outlined in Table 2) on synthetic datasets encompassing individuals of five ancestry groups. We created two compendia of synthetic datasets with case-to-control ratio of 1:1 (SD) and 1:4 (SD*), respectively. Using synthetic data enables us to test the generalizability of our findings across a broad spectrum of conditions characterized by ancestry, heritability, and shift of genotype–phenotype relationship between ancestry groups (represented by the parameter ρ). We evaluated the machine learning performance on the synthetic datasets using AUROC, AUPR, Tjur’s *R*^2^, PPV, and NPV (Table 5 and Fig. 2, Table S9–S17 and Fig. S4–S6).

**Table 5:** Multi-ancestral machine learning experiments on synthetic dataset compendium SD

Synthetic datasets				Area under ROC curve (LR, DL)						
**ID**	**DDP**	${{\varvec{h}}}^{2}$	${\varvec{\rho}}$	**Mix0**	**Mix1**	**Mix2**	**Ind1**	**Ind2**	**NT**	**TL**
SD1	AMR	0.50	0.80	0.77, 0.78	0.77, 0.79	0.73, 0.73	0.77, 0.79	0.69, 0.70	0.71, 0.71	0.70, 0.74
SD2	SAS	0.50	0.77	0.77, 0.79	0.78, 0.79	0.72, 0.73	0.78, 0.79	0.69, 0.70	0.71, 0.70	0.74, 0.74
SD3	EAS	0.50	0.58	0.75, 0.77	0.76, 0.78	0.67, 0.70	0.77, 0.77	0.69, 0.66	0.62, 0.56	0.67, 0.72
SD4	AFR	0.50	0.54	0.76, 0.77	0.77, 0.78	0.69, 0.72	0.77, 0.78	0.70, 0.69	0.65, 0.64	0.69, 0.74
SD5	AMR	0.25	0.80	0.65, 0.66	0.65, 0.66	0.63, 0.62	0.65, 0.65	0.57, 0.57	0.62, 0.62	0.63, 0.64
SD6	SAS	0.25	0.77	0.64, 0.66	0.65, 0.66	0.62, 0.62	0.64, 0.66	0.58, 0.59	0.61, 0.61	0.60, 0.64
SD7	EAS	0.25	0.58	0.62, 0.64	0.63, 0.64	0.59, 0.60	0.63, 0.65	0.59, 0.61	0.56, 0.55	0.54, 0.62
SD8	AFR	0.25	0.54	0.65, 0.65	0.65, 0.66	0.62, 0.61	0.65, 0.66	0.60, 0.61	0.60, 0.59	0.57, 0.64
SD9	AMR	0.50	0.80	0.77, 0.78	0.77, 0.79	0.73, 0.73	0.77, 0.79	0.69, 0.70	0.71, 0.71	0.70, 0.74
SD10	SAS	0.50	0.74	0.76, 0.77	0.77, 0.78	0.72, 0.73	0.77, 0.78	0.68, 0.69	0.70, 0.68	0.66, 0.75
SD11	EAS	0.50	0.42	0.75, 0.76	0.77, 0.78	0.64, 0.64	0.77, 0.78	0.67, 0.68	0.60, 0.60	0.63, 0.70
SD12	AFR	0.50	0.36	0.74, 0.76	0.76, 0.77	0.61, 0.66	0.77, 0.77	0.68, 0.67	0.56, 0.57	0.59, 0.71
SD13	AMR	0.25	0.80	0.65, 0.66	0.65, 0.66	0.63, 0.62	0.65, 0.65	0.57, 0.57	0.62, 0.62	0.63, 0.64
SD14	SAS	0.25	0.74	0.64, 0.64	0.65, 0.65	0.62, 0.62	0.64, 0.65	0.57, 0.57	0.60, 0.54	0.60, 0.62
SD15	EAS	0.25	0.42	0.64, 0.65	0.65, 0.66	0.57, 0.58	0.65, 0.65	0.58, 0.56	0.54, 0.52	0.55, 0.61
SD16	AFR	0.25	0.36	0.61, 0.62	0.62, 0.63	0.54, 0.55	0.63, 0.63	0.57, 0.58	0.52, 0.51	0.56, 0.61

*LR* Logistic regression, *DL* Deep learning, *SD* Synthetic dataset compendium with a case-to-control ratio of 1:1, *DDP* Data-disadvantaged population, *AFR* African, *AMR* Admixed American, *EAS* East Asian, *SAS* South Asian. Mix0, Mix1, Mix2, Ind1, Ind2, NT, and TL are the machine learning experiments outlined in Table 2; SD9 and SD13 are the same as SD1 and SD5 and are included here for comparison

As expected, heritability (${h}^{2}$) influences prediction performance in all experiments, with higher heritability linked to more accurate predictions. Nevertheless, our key observations remain consistent at different levels of heritability (${h}^{2}=0.5\ \text{and}\ {h}^{2}=0.25$). The parameter $\rho$, representing the cross-ancestry correlation of the genetic effects, is determined based on genetic distance between ancestry groups (detailed in the Methods section). Therefore, $\rho$ indicates the degree of data distribution shifts between the EUR population and DDPs. In comparing machine learning model performance across synthetic datasets, we observed statistically significant performance disparity gaps between EUR and DDPs in both mixture and independent learning. There were also significant improvements with DL-based transfer learning, unlike with LR-based transfer learning, where improvements were generally absent. Moreover, DL-based transfer learning consistently outperformed LR-based transfer learning (Table 6, Table S18). Overall, the key observations are robust across the synthetic datasets representing various levels of heritability and data distribution shifts among ancestry groups.

**Table 6:** Disparity detection and mitigation in multi-ancestral machine learning using synthetic datasets

Key observations	Comparison	Mean difference in AUROC	
		**SD**	SD^*^
Performance disparity gap between EUR and DDPs in mixture and independent learning	Mix1_LR vs Mix2_LR	**0.06**	**0.07**
	Mix1_DL vs Mix2_DL	**0.06**	**0.06**
	Ind1_LR vs Ind2_LR	**0.08**	**0.07**
	Ind1_DL vs Ind2_DL	**0.08**	**0.07**
Improvement from DL-based transfer learning	TL_DL vs Mix2_DL	**0.02**	**0.03**
	TL_DL vs Ind2_DL	**0.04**	**0.04**
	TL_DL vs NT_DL	**0.07**	**0.07**
Improvement from LR-based transfer learning	TL_LR vs Mix2_LR	-0.02	-0.02
	TL_LR vs Ind2_LR	-0.01	-0.02
	TL_LR vs NT_LR	0.01	0.00
Performance difference between DL- and LR-based transfer learning	TL_DL vs TL_LR	**0.05**	**0.07**

The statistically significant (*p*-value less than 0.05) performance differences are highlighted using bold font. *LR* Logistic regression, *DL* Deep learning, *SD* Synthetic dataset compendium with a case-to-control ratio of 1:1; SD^*^: Synthetic dataset compendium with a case-to-control ratio of 1:4; Mix0, Mix1, Mix2, Ind1, Ind2, NT, and TL are the machine learning experiments outlined in Table 2

We also compared the performance of DL and LR models in each of the seven types of experiments, as outlined in Table 2, using the two compendia of synthetic datasets (Table S19). In all mixture learning experiments (Mix0, Mix1, and Mix2) and one independent learning experiment (Ind1), DL outperformed LR, albeit by a small yet statistically significant (*p*-value < 0.05) margin, with a mean AUROC difference (DL-LR) of 0.01 to 0.02. No significant performance differences were observed in the Ind2 and naïve transfer learning (NT) experiments. The advantage of DL over LR was largest in transfer learning, where DL-based transfer learning outperformed by a mean AUROC difference of 0.05 to 0.07. We also used AUPR, Tjur’s *R*^2^, PPV, and NPV as metrics to compare the performance of DL and LR models. While the relative performance of DL and LR vary in the mixture and independent learning scenarios, the advantage of DL over LR was consistently large (ranging from 0.04 to 0.10) and statistically significant (*p*-value < 0.05) in transfer learning (Table S19). Furthermore, we compared the performance of DL and LR models across all seven types of experiments. The performance differences in AUROC, AUPR, Tjur’s *R*^2^, PPV, and NPV are statistically significant (*p*-value < 0.05), except for the differences in the AUPR and Tjur’s *R*^2^ metrics for the SD compendium.

**Fig. 3:**
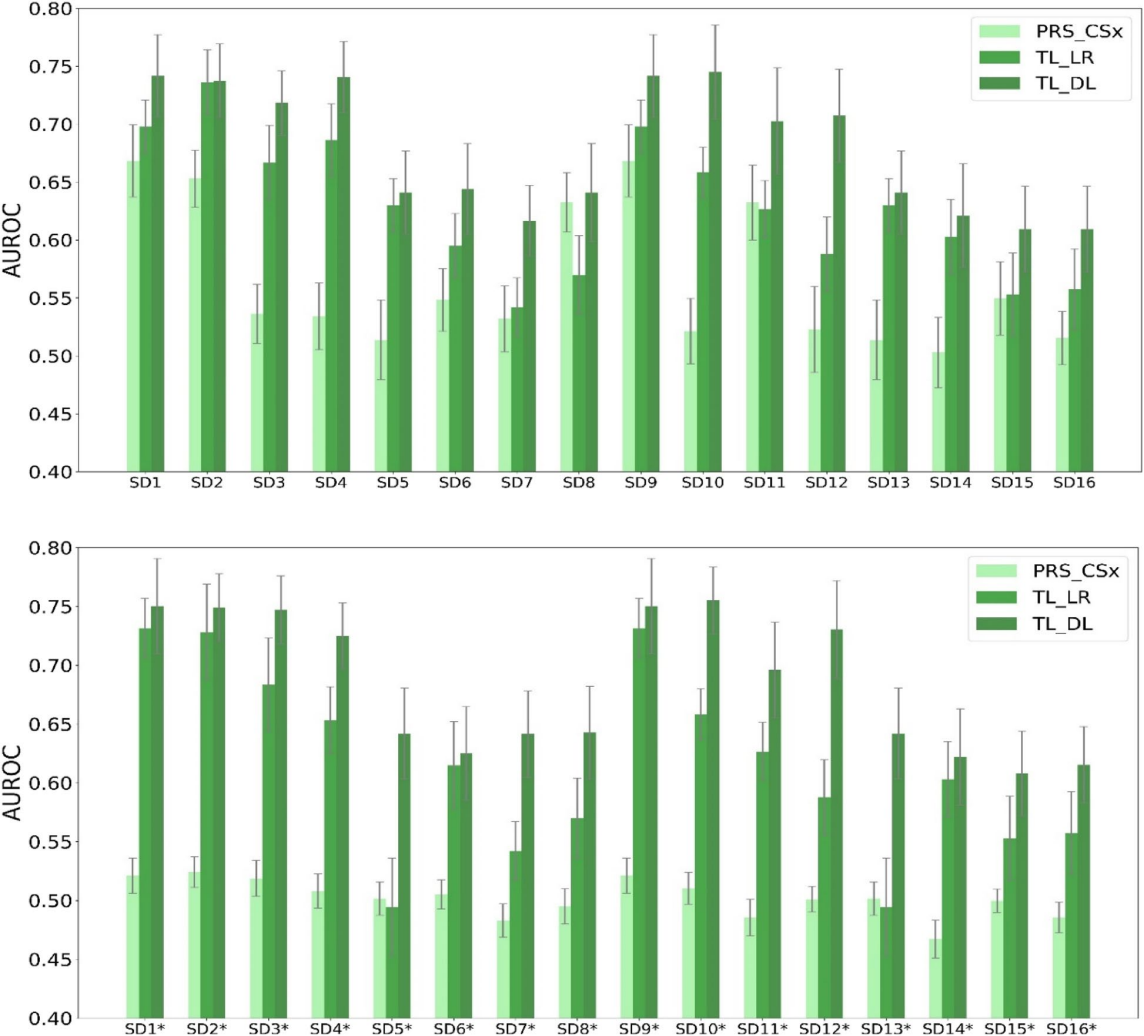
Performance of PRS_CSx, TL_LR, and TL_DL across the synthetic datasets. The error bar of each column represents the standard deviation of the machine learning model performance (AUROC) of 20 independent runs. TL_LR, logistic regression-based transfer learning; TL_DL, deep learning-based transfer learning

In a broader sense, PRS-CSx [16] can also be viewed as a form of transfer learning, as it applies knowledge from a source domain (EUR) to improve prediction accuracy in a target domain (DDP). As an extension of the PRS-CS (Polygenic Risk Score using Continuous Shrinkage) method [77], PRS-CSx retains the linear framework while accounting for cross-population differences in allele frequencies and linkage disequilibrium structures. Since the current form of PRS-CSx does not incorporate clinical features into the phenotype prediction model, it is not directly applicable to the real datasets used in this study. We compared the performance of PRS-CSx with LR- and DL-based transfer learning on the two compendia of synthetic datasets and found that DL-based transfer learning showed the best performance on all of them (Fig. 3), confirming that DL models are more amenable to transfer learning in the context of multi-ancestral genomic prediction.

## Discussion

Precision medicine increasingly relies on the predictive power of machine learning, with biomedical data forming the crucial foundation for developing high-quality models. Traditional polygenic models, primarily based on linear frameworks [78–85], are often inadequate for accurate, individualized disease prediction. This is mainly attributable to two factors: firstly, complex diseases result from an interplay of genetic, environmental, and lifestyle factors, not solely genetic determinants; and secondly, linear polygenic models lack the expressive power and model capacity to capture the non-linear, non-additive interactions inherent in the complex genotype–phenotype relationship. The inability to model non-additive genetic interactions significantly reduces the accuracy of polygenic prediction [86]. Recently, deep learning and other machine learning models that excel at handling complex nonlinearity have been used for genomic prediction of diseases [87, 88]. These models outperformed traditional polygenic prediction models in many applications [89–93]. In this study, we compared the performance of deep learning (DL) and logistic regression (LR) models across various multi-ancestral learning schemes. While DL outperformed LR in many, but not all, mixture and independent learning experiments, DL-based transfer learning consistently outperformed LR-based transfer learning across all the real and synthetic datasets. This finding suggests that DL-based genomic prediction models are especially adept at transfer learning, even though they do not always outperform LR-based models under commonly used multi-ancestral machine learning schemes, such as mixture and independent learning.

Genomic data inequality significantly impedes the development of equitable machine learning in precision medicine, thereby posing substantial health risks to populations with limited data representation. Currently, fairness-aware machine learning [94] often relies on various ad hoc constraints or penalty terms within loss functions and training processes to enforce performance parity across different subpopulations. However, this approach leads to a fairness-accuracy tradeoff, a predicament where ensuring fairness may come at the cost of reduced accuracy for one or more subpopulations [95, 96]. A significant advantage of the deep transfer learning approach is that it is not subject to such fairness-accuracy tradeoffs. It can improve the performance of AI models for the data-disadvantaged populations by consulting but not affecting the model for the data-rich population.

Multi-ancestral machine learning can be viewed as a multi-objective optimization problem, with the goal of maximizing prediction accuracy for all ancestry groups involved in a learning task. Pareto improvement [27], a concept originated from economics, is important and relevant to multi-objective optimization because it inherently recognizes the presence of multiple, often competing, objectives and facilitates the identification of solutions that improve at least one objective without detriment to others [97, 98]. It is worth noting that Pareto improvement reflects the foundational principle in medical ethics of “first, do no harm (primum non nocere)”, traditionally linked to the Hippocratic Oath taken by healthcare professionals. Recently, this principle has been extended to guide the development, deployment, and use of artificial intelligence technologies [99, 100]. Pareto improvement is particularly beneficial for advancing equitable genomic medicine, as it enhances disease prediction for data-disadvantaged populations without adversely affecting the outcomes for other populations.

## Conclusions

This study shows that deep transfer learning provides a Pareto improvement towards equitable multi-ancestral machine learning for clinico-genomic prediction of diseases, as it improves prediction accuracy for data-disadvantaged populations without compromising accuracy for other populations. Machine learning experiments using synthetic data confirm that this improvement is consistent across various levels of heritability and data distribution shifts among ancestry groups.

### Supplementary Information

Additional file 1: Supplementary tables Table S1-S19 and supplementary figures Fig.S1-Fig.S6. 

## Data Availability

The genotype–phenotype datasets are available from the dbGaP database (https://www.ncbi.nlm.nih.gov/gap/) [101] under the study accession numbers phs001273.v3.p2 [102], phs001391.v1.p1 [103], phs000496.v1.p1 [104], and phs000372.v1.p1 [105]. The simulated genotype data are available from the Harvard Dataverse (10.7910/DVN/COXHAP) [106]. The synthetic datasets are available from https://figshare.com/articles/media/TLGP_GM/25377532 [107]. The source code is available from https://github.com/ai4pm/TLGP [108].
