# Supplementary material for: Optimizing clinico-genomic disease prediction across ancestries: a machine learning strategy with Pareto improvement
Source: Genome Med. 2024 Jun 4;16:76. doi: 10.1186/s13073-024-01345-0 (PMC11149372; doi:10.1186/s13073-024-01345-0)
Supplement: Supplementary file 1 — Additional file 1: Supplementary tables Table S1-S19 and supplementary figures Fig.S1-Fig.S6. [file 13073_2024_1345_MOESM1_ESM.pdf]

Supplemental information

Table S1 Multi-ancestral clinico-genomic prediction of diseases (AUPR)

Datasets		Area Under Precision-Recall Curve (LR, DL)						
**Disease**	**DDP**	**Mix0**	**Mix1**	**Mix2**	**Ind1**	**Ind2**	**NT**	**TL**
Lung Cancer	East Asian	0.69, 0.73	0.69, 0.73	0.70, 0.71	0.67, 0.72	0.67, 0.65	0.69, 0.67	0.65, 0.75
Prostate Cancer	African American	0.78, 0.78	0.79, 0.79	0.56, 0.53	0.79, 0.79	0.52, 0.50	0.48, 0.52	0.50, 0.56
Alzheimer’s Disease	Latin American	0.79, 0.79	0.83, 0.83	0.59, 0.59	0.83, 0.83	0.59, 0.59	0.55, 0.55	0.53, 0.61
Alzheimer’s Disease	African American	0.82, 0.82	0.85, 0.85	0.55, 0.55	0.85, 0.85	0.53, 0.53	0.52, 0.52	0.54, 0.61

LR: Logistic regression; **DL:** Deep learning; **DDP**: Data-disadvantaged population. **Mix0, Mix1, Mix2, Ind1, Ind2, NT, and TL** are the machine learning experiments outlined in Table 2.

Table S2 Multi-ancestral clinico-genomic prediction of diseases (Tjur’s R^2)

Datasets		Tjur’s R^2 (LR, DL)						
**Disease**	**DDP**	**Mix0**	**Mix1**	**Mix2**	**Ind1**	**Ind2**	**NT**	**TL**
Lung Cancer	East Asian	0.11, 0.11	0.11, 0.12	0.02, 0.02	0.12, 0.12	0.02, 0.00	0.05, 0.00	0.04, 0.10
Prostate Cancer	African American	0.15, 0.13	0.16, 0.13	0.06, 0.06	0.16, 0.13	0.06, 0.03	0.05, 0.06	0.05, 0.09
Alzheimer’s Disease	Latin American	0.09, 0.12	0.08, 0.11	0.03, 0.04	0.08, 0.12	0.01, 0.02	0.03, 0.02	0.02, 0.06
Alzheimer’s Disease	African American	0.10, 0.12	0.09, 0.11	0.03, 0.02	0.09, 0.12	0.02, 0.01	0.02, 0.04	0.03, 0.07

LR: Logistic regression; **DL:** Deep learning; **DDP**: Data-disadvantaged population. **Mix0, Mix1, Mix2, Ind1, Ind2, NT, and TL** are the machine learning experiments outlined in Table 2.

Table S3 Disparity detection and mitigation in multi-ancestral clinico-genomic prediction (AUPR and Tjur's R^2)

Key observations	Comparison	Mean Difference in AUPR				Mean Difference in Tjur's R^2			
		LC	PC	AD1	AD2	LC	PC	AD1	AD2
Performance disparity gap between EUR and DDPs in mixture and independent learning	Mix1_LR vs Mix2_LR	-0.01	**0.24**	**0.24**	**0.30**	**0.09**	**0.10**	**0.05**	**0.06**
	Mix1_DL vs Mix2_DL	**0.02**	**0.26**	**0.24**	**0.30**	**0.10**	**0.07**	**0.07**	**0.09**
	Ind1_LR vs Ind2_LR	-0.01	**0.27**	**0.23**	**0.32**	**0.10**	**0.10**	**0.07**	**0.07**
	Ind1_DL vs Ind2_DL	**0.08**	**0.29**	**0.23**	**0.32**	**0.12**	**0.10**	**0.10**	**0.11**
Improvement from DL-based transfer learning	TL_DL vs Mix2_DL	**0.03**	**0.03**	**0.02**	**0.06**	**0.08**	**0.03**	**0.02**	**0.05**
	TL_DL vs Ind2_DL	**0.10**	**0.06**	0.02	**0.08**	**0.10**	**0.06**	**0.04**	**0.06**
	TL_DL vs NT_DL	**0.07**	**0.04**	**0.06**	**0.09**	**0.10**	**0.03**	**0.03**	**0.03**
Improvement from LR-based transfer learning	TL_LR vs Mix2_LR	-0.05	-0.05	-0.06	0.00	0.01	-0.01	-0.01	0.01
	TL_LR vs Ind2_LR	-0.02	-0.01	-0.07	0.02	0.02	-0.01	-0.01	0.01
	TL_LR vs NT_LR	-0.04	**0.02**	-0.03	**0.02**	-0.01	0.00	0.00	0.01
Performance difference between DL- and LR-based transfer learning	TL_DL vs TL_LR	**0.09**	**0.06**	**0.08**	**0.06**	**0.06**	**0.04**	**0.04**	**0.03**

The statistically significant (p-value less than 0.05) performance differences are highlighted using bold font. **LC, PC, AD1, and AD2** are the clinico-genomic datasets; **LC**: Lung cancer (European and East Asian populations); **PC**: Prostate cancer (European and African American populations); **AD1**: Alzheimer's disease (European and Latin American populations); **AD2**: Alzheimer's disease (European and African American populations); **LR:** Logistic regression; **DL:** Deep learning; **Mix0, Mix1, Mix2, Ind1, Ind2, NT, and TL** are the machine learning experiments outlined in Table 2.

Table S4 Multi-ancestral clinico-genomic prediction of diseases (Sensitivity)

Datasets		Sensitivity (LR, DL)						
**Disease**	**DDP**	**Mix0**	**Mix1**	**Mix2**	**Ind1**	**Ind2**	**NT**	**TL**
Lung Cancer	East Asian	0.65, 0.72	0.63, 0.71	0.47, 0.48	0.66, 0.72	0.54, 0.54	0.52, 0.55	0.57, 0.62
Prostate Cancer	African American	0.64, 0.64	0.65, 0.64	0.46, 0.53	0.64, 0.66	0.51, 0.55	0.56, 0.55	0.57, 0.64
Alzheimer’s Disease	Latin American	0.51, 0.53	0.56, 0.58	0.45, 0.51	0.57, 0.58	0.46, 0.45	0.37, 0.53	0.48, 0.60
Alzheimer’s Disease	African American	0.58, 0.57	0.65, 0.63	0.53, 0.52	0.64, 0.63	0.48, 0.53	0.56, 0.52	0.52, 0.65

LR: Logistic regression; **DL:** Deep learning; **DDP**: Data-disadvantaged population. **Mix0, Mix1, Mix2, Ind1, Ind2, NT, and TL** are the machine learning experiments outlined in Table 2.

Table S5 Multi-ancestral clinico-genomic prediction of diseases (Specificity)

Datasets		Specificity (LR, DL)						
**Disease**	**DDP**	**Mix0**	**Mix1**	**Mix2**	**Ind1**	**Ind2**	**NT**	**TL**
Lung Cancer	East Asian	0.60, 0.61	0.68, 0.70	0.58, 0.60	0.71, 0.73	0.55, 0.59	0.61, 0.61	0.60, 0.68
Prostate Cancer	African American	0.69, 0.69	0.69, 0.70	0.58, 0.56	0.70, 0.71	0.60, 0.58	0.54, 0.59	0.56, 0.67
Alzheimer’s Disease	Latin American	0.76, 0.81	0.74, 0.80	0.61, 0.61	0.74, 0.80	0.63, 0.64	0.55, 0.57	0.60, 0.69
Alzheimer’s Disease	African American	0.77, 0.76	0.72, 0.72	0.61, 0.60	0.74, 0.74	0.57, 0.58	0.57, 0.62	0.62, 0.68

LR: Logistic regression; **DL:** Deep learning; **DDP**: Data-disadvantaged population. **Mix0, Mix1, Mix2, Ind1, Ind2, NT, and TL** are the machine learning experiments outlined in Table 2.

Table S6 Multi-ancestral clinico-genomic prediction of diseases (Prevalence-adjusted PPV)

Datasets		Prevalence-adjusted PPV (LR, DL)					
**Disease**	**DDP**	**Mix1**	**Mix2**	**Ind1**	**Ind2**	**NT**	**TL**
Alzheimer’s Disease	Latin American	0.20, 0.26	0.14, 0.16	0.20, 0.27	0.15, 0.15	0.10, 0.14	0.14, 0.21
Alzheimer’s Disease	African American	0.21, 0.22	0.18, 0.18	0.22, 0.22	0.15, 0.17	0.17, 0.18	0.18, 0.25

LR: Logistic regression; **DL:** Deep learning; **DDP**: Data-disadvantaged population. **Mix1, Mix2, Ind1, Ind2, NT, and TL** are the machine learning experiments outlined in Table 2.

Table S7 Multi-ancestral clinico-genomic prediction of diseases (Prevalence-adjusted NPV)

Datasets		Prevalence-adjusted NPV (LR, DL)					
**Disease**	**DDP**	**Mix1**	**Mix2**	**Ind1**	**Ind2**	**NT**	**TL**
Alzheimer’s Disease	Latin American	0.94, 0.94	0.89, 0.90	0.94, 0.94	0.89, 0.89	0.86, 0.90	0.89, 0.92
Alzheimer’s Disease	African American	0.94, 0.95	0.89, 0.89	0.95, 0.95	0.87, 0.89	0.89, 0.89	0.89, 0.93

LR: Logistic regression; **DL:** Deep learning; **DDP**: Data-disadvantaged population. **Mix1, Mix2, Ind1, Ind2, NT, and TL** are the machine learning experiments outlined in Table 2.

Table S8 Disparity detection and mitigation in multi-ancestral clinico-genomic prediction (Prevalence-adjusted PPV and NPV)

Key observations	Comparison	Mean Difference in Prevalence-adjusted PPV		Mean Difference in Prevalence-adjusted NPV	
		AD1	AD2	AD1	AD2
Performance disparity gap between EUR and DDPs in mixture and independent learning	Mix1_LR vs Mix2_LR	**0.06**	**0.03**	**0.05**	**0.05**
	Mix1_DL vs Mix2_DL	**0.10**	**0.04**	**0.04**	**0.06**
	Ind1_LR vs Ind2_LR	**0.06**	**0.07**	**0.04**	**0.07**
	Ind1_DL vs Ind2_DL	**0.12**	**0.04**	**0.05**	**0.06**
Improvement from DL-based transfer learning	TL_DL vs Mix2_DL	**0.06**	**0.07**	**0.03**	**0.04**
	TL_DL vs Ind2_DL	**0.06**	**0.07**	**0.03**	**0.04**
	TL_DL vs NT_DL	**0.07**	**0.07**	**0.03**	**0.03**
Improvement from LR-based transfer learning	TL_LR vs Mix2_LR	0.00	0.01	**0.00**	0.00
	TL_LR vs Ind2_LR	0.00	0.03	0.00	0.01
	TL_LR vs NT_LR	**0.04**	0.01	**0.03**	0.00
Performance difference between DL- and LR-based transfer learning	TL_DL vs TL_LR	**0.07**	**0.06**	**0.03**	**0.04**

The statistically significant (p-value less than 0.05) performance differences are highlighted using bold font. **LC, PC, AD1, and AD2** are the clinico-genomic datasets; **LC**: Lung cancer (European and East Asian populations); **PC**: Prostate cancer (European and African American populations); **AD1**: Alzheimer's disease (European and Latin American populations); **AD2**: Alzheimer's disease (European and African American populations); **LR:** Logistic regression; **DL:** Deep learning; **Mix0, Mix1, Mix2, Ind1, Ind2, NT, and TL** are the machine learning experiments outlined in Table 2.

Table S9 Multi-ancestral machine learning experiments on synthetic dataset compendium SD* (AUROC)

Synthetic Datasets				Area Under ROC Curve (LR, DL)						
**ID**	**DDP**	h^2	ρ	**Mix0**	**Mix1**	**Mix2**	**Ind1**	**Ind2**	**NT**	**TL**
SD1*	AMR	0.50	0.80	0.76, 0.77	0.76, 0.78	0.73, 0.73	0.77, 0.77	0.71, 0.70	0.70, 0.71	0.73, 0.75
SD2*	SAS	0.50	0.77	0.76, 0.77	0.76, 0.78	0.73, 0.73	0.76, 0.78	0.71, 0.70	0.72, 0.69	0.73, 0.75
SD3*	EAS	0.50	0.58	0.76, 0.76	0.77, 0.77	0.72, 0.70	0.77, 0.77	0.72, 0.71	0.66, 0.56	0.68, 0.75
SD4*	AFR	0.50	0.54	0.76, 0.76	0.77, 0.77	0.68, 0.71	0.78, 0.78	0.71, 0.72	0.64, 0.63	0.65, 0.73
SD5*	AMR	0.25	0.80	0.63, 0.65	0.64, 0.66	0.54, 0.61	0.64, 0.66	0.55, 0.58	0.52, 0.62	0.49, 0.64
SD6*	SAS	0.25	0.77	0.65, 0.65	0.66, 0.65	0.60, 0.61	0.65, 0.65	0.53, 0.59	0.60, 0.61	0.61, 0.63
SD7*	EAS	0.25	0.58	0.66, 0.67	0.67, 0.67	0.61, 0.61	0.66, 0.67	0.63, 0.62	0.63, 0.56	0.54, 0.64
SD8*	AFR	0.25	0.54	0.60, 0.65	0.60, 0.66	0.56, 0.63	0.62, 0.65	0.55, 0.60	0.52, 0.60	0.57, 0.64
SD9*	AMR	0.50	0.80	0.76, 0.77	0.76, 0.78	0.73, 0.73	0.77, 0.77	0.71, 0.70	0.70, 0.71	0.73, 0.75
SD10*	SAS	0.50	0.74	0.78, 0.77	0.77, 0.78	0.72, 0.73	0.77, 0.78	0.67, 0.69	0.69, 0.67	0.66, 0.76
SD11*	EAS	0.50	0.42	0.73, 0.76	0.74, 0.78	0.68, 0.67	0.74, 0.78	0.68, 0.66	0.62, 0.61	0.63, 0.70
SD12*	AFR	0.50	0.36	0.79, 0.79	0.79, 0.78	0.62, 0.66	0.79, 0.78	0.67, 0.69	0.58, 0.56	0.59, 0.73
SD13*	AMR	0.25	0.80	0.63, 0.65	0.64, 0.66	0.54, 0.61	0.64, 0.66	0.55, 0.58	0.52, 0.62	0.49, 0.64
SD14*	SAS	0.25	0.74	0.62, 0.65	0.60, 0.65	0.60, 0.60	0.60, 0.65	0.60, 0.59	0.60, 0.58	0.60, 0.62
SD15*	EAS	0.25	0.42	0.65, 0.64	0.66, 0.65	0.57, 0.58	0.66, 0.65	0.57, 0.56	0.55, 0.52	0.55, 0.61
SD16*	AFR	0.25	0.36	0.65, 0.64	0.66, 0.67	0.57, 0.59	0.66, 0.66	0.57, 0.60	0.53, 0.57	0.56, 0.62

LR: Logistic regression; **DL:** Deep learning; **SD***: Synthetic dataset compendium with a case-to-control ratio of 1:4; **DDP**: Data-disadvantaged population; **AFR**: African; **AMR**: Admixed American; **EAS**: East Asian; **SAS**: South Asian. **Mix0, Mix1, Mix2, Ind1, Ind2, NT, and TL** are the machine learning experiments outlined in Table 2; SD9* and SD13* are same as SD1* and SD5* and are included here for comparison.

Table S10 Multi-ancestral machine learning experiments on synthetic data compendium SD (AUPR)

Synthetic Datasets				Area Under Precision-Recall Curve (LR, DL)						
**ID**	**DDP**	h^2	ρ	**Mix0**	**Mix1**	**Mix2**	**Ind1**	**Ind2**	**NT**	**TL**
SD1	AMR	0.50	0.80	0.76, 0.77	0.78, 0.78	0.69, 0.73	0.77, 0.78	0.65, 0.69	0.68, 0.67	0.67, 0.73
SD2	SAS	0.50	0.77	0.79, 0.77	0.80, 0.78	0.74, 0.73	0.80, 0.78	0.67, 0.68	0.73, 0.65	0.72, 0.75
SD3	EAS	0.50	0.58	0.74, 0.76	0.76, 0.77	0.67, 0.70	0.76, 0.77	0.71, 0.70	0.62, 0.54	0.65, 0.72
SD4	AFR	0.50	0.54	0.76, 0.77	0.77, 0.78	0.69, 0.71	0.77, 0.78	0.69, 0.63	0.65, 0.58	0.68, 0.73
SD5	AMR	0.25	0.80	0.66, 0.65	0.66, 0.65	0.64, 0.63	0.65, 0.65	0.61, 0.59	0.61, 0.59	0.64, 0.65
SD6	SAS	0.25	0.77	0.66, 0.64	0.66, 0.66	0.63, 0.63	0.66, 0.64	0.61, 0.59	0.62, 0.59	0.61, 0.64
SD7	EAS	0.25	0.58	0.61, 0.62	0.63, 0.62	0.53, 0.60	0.63, 0.62	0.60, 0.61	0.50, 0.53	0.55, 0.62
SD8	AFR	0.25	0.54	0.65, 0.64	0.66, 0.65	0.60, 0.61	0.66, 0.64	0.61, 0.59	0.57, 0.57	0.58, 0.62
SD9	AMR	0.50	0.80	0.76, 0.77	0.78, 0.78	0.69, 0.73	0.77, 0.78	0.65, 0.69	0.68, 0.67	0.67, 0.73
SD10	SAS	0.50	0.74	0.77, 0.76	0.78, 0.77	0.69, 0.73	0.78, 0.77	0.69, 0.70	0.66, 0.66	0.68, 0.74
SD11	EAS	0.50	0.42	0.75, 0.76	0.79, 0.78	0.61, 0.65	0.79, 0.78	0.66, 0.66	0.59, 0.59	0.61, 0.68
SD12	AFR	0.50	0.36	0.73, 0.75	0.76, 0.77	0.59, 0.65	0.76, 0.77	0.70, 0.70	0.54, 0.56	0.58, 0.72
SD13	AMR	0.25	0.80	0.66, 0.65	0.66, 0.65	0.64, 0.63	0.65, 0.65	0.61, 0.59	0.61, 0.59	0.64, 0.65
SD14	SAS	0.25	0.74	0.65, 0.64	0.66, 0.64	0.60, 0.61	0.66, 0.65	0.57, 0.58	0.60, 0.55	0.61, 0.62
SD15	EAS	0.25	0.42	0.63, 0.64	0.65, 0.64	0.55, 0.59	0.66, 0.64	0.61, 0.60	0.51, 0.52	0.56, 0.62
SD16	AFR	0.25	0.36	0.61, 0.61	0.63, 0.62	0.57, 0.57	0.63, 0.62	0.59, 0.56	0.56, 0.51	0.56, 0.60

LR: Logistic regression; **DL:** Deep learning; **SD**: Synthetic dataset compendium with a case-to-control ratio of 1:1; **DDP**: Data-disadvantaged population; **AFR**: African; **AMR**: Admixed American; **EAS**: East Asian; **SAS**: South Asian. **Mix0, Mix1, Mix2, Ind1, Ind2, NT, and TL** are the machine learning experiments outlined in Table 2; SD9 and SD13 are same as SD1 and SD5 and are included here for comparison.

Table S11 Multi-ancestral machine learning experiments on synthetic data compendium SD* (AUPR)

Synthetic Datasets				Area Under Precision-Recall Curve (LR, DL)						
ID	DDP	h^2	ρ	Mix0	Mix1	Mix2	Ind1	Ind2	NT	TL
SD1*	AMR	0.50	0.80	0.42, 0.42	0.43, 0.45	0.41, 0.43	0.44, 0.42	0.36, 0.41	0.37, 0.36	0.40, 0.47
SD2*	SAS	0.50	0.77	0.44, 0.43	0.45, 0.46	0.40, 0.43	0.46, 0.43	0.42, 0.39	0.37, 0.36	0.38, 0.48
SD3*	EAS	0.50	0.58	0.48, 0.50	0.50, 0.51	0.41, 0.46	0.53, 0.51	0.37, 0.44	0.33, 0.32	0.22, 0.51
SD4*	AFR	0.50	0.54	0.51, 0.52	0.57, 0.54	0.35, 0.40	0.59, 0.54	0.38, 0.32	0.34, 0.26	0.34, 0.42
SD5*	AMR	0.25	0.80	0.42, 0.42	0.43, 0.45	0.41, 0.43	0.44, 0.42	0.36, 0.41	0.37, 0.36	0.40, 0.47
SD6*	SAS	0.25	0.77	0.32, 0.31	0.33, 0.32	0.29, 0.29	0.31, 0.31	0.24, 0.24	0.28, 0.28	0.27, 0.30
SD7*	EAS	0.25	0.58	0.34, 0.36	0.35, 0.36	0.30, 0.34	0.35, 0.37	0.29, 0.33	0.27, 0.22	0.21, 0.35
SD8*	AFR	0.25	0.54	0.28, 0.27	0.29, 0.29	0.27, 0.26	0.29, 0.28	0.23, 0.25	0.25, 0.20	0.24, 0.27
SD9*	AMR	0.50	0.80	0.42, 0.42	0.43, 0.45	0.41, 0.43	0.44, 0.42	0.36, 0.41	0.37, 0.36	0.40, 0.47
SD10*	SAS	0.50	0.74	0.50, 0.51	0.53, 0.53	0.38, 0.43	0.53, 0.52	0.33, 0.33	0.34, 0.37	0.37, 0.47
SD11*	EAS	0.50	0.42	0.39, 0.41	0.41, 0.43	0.35, 0.38	0.41, 0.41	0.37, 0.39	0.30, 0.30	0.19, 0.45
SD12*	AFR	0.50	0.36	0.49, 0.50	0.55, 0.53	0.31, 0.35	0.57, 0.52	0.35, 0.34	0.27, 0.27	0.26, 0.38
SD13*	AMR	0.25	0.80	0.42, 0.42	0.43, 0.45	0.41, 0.43	0.44, 0.42	0.36, 0.41	0.37, 0.36	0.40, 0.47
SD14*	SAS	0.25	0.74	0.29, 0.29	0.30, 0.33	0.28, 0.27	0.31, 0.32	0.28, 0.29	0.30, 0.28	0.36, 0.37
SD15*	EAS	0.25	0.42	0.30, 0.32	0.31, 0.33	0.25, 0.29	0.31, 0.32	0.26, 0.25	0.23, 0.24	0.24, 0.31
SD16*	AFR	0.25	0.36	0.30, 0.32	0.31, 0.33	0.30, 0.28	0.31, 0.33	0.29, 0.27	0.27, 0.28	0.29, 0.31

LR: Logistic regression; **DL:** Deep learning; **SD***: Synthetic dataset compendium with a case-to-control ratio of 1:4; **DDP**: Data-disadvantaged population; **AFR**: African; **AMR**: Admixed American; **EAS**: East Asian; **SAS**: South Asian. **Mix0, Mix1, Mix2, Ind1, Ind2, NT, and TL** are the machine learning experiments outlined in Table 2; SD9* and SD13* are same as SD1* and SD5* and are included here for comparison.

Table S12 Multi-ancestral machine learning experiments on synthetic data compendium SD (Tjur's R^2)

Synthetic Datasets				Tjur's R^2 (LR, DL)						
**ID**	**DDP**	h^2	ρ	**Mix0**	**Mix1**	**Mix2**	**Ind1**	**Ind2**	**NT**	**TL**
SD1	AMR	0.50	0.80	0.26, 0.24	0.27, 0.25	0.19, 0.20	0.27, 0.26	0.23, 0.23	0.18, 0.23	0.23, 0.28
SD2	SAS	0.50	0.77	0.28, 0.25	0.28, 0.26	0.23, 0.20	0.29, 0.25	0.20, 0.26	0.25, 0.26	0.27, 0.27
SD3	EAS	0.50	0.58	0.23, 0.22	0.24, 0.24	0.17, 0.15	0.26, 0.24	0.20, 0.20	0.10, 0.07	0.20, 0.26
SD4	AFR	0.50	0.54	0.24, 0.23	0.25, 0.24	0.18, 0.17	0.27, 0.24	0.19, 0.17	0.14, 0.21	0.21, 0.25
SD5	AMR	0.25	0.80	0.11, 0.10	0.11, 0.10	0.07, 0.08	0.11, 0.09	0.06, 0.06	0.07, 0.07	0.08, 0.11
SD6	SAS	0.25	0.77	0.11, 0.09	0.11, 0.10	0.08, 0.08	0.12, 0.09	0.08, 0.08	0.08, 0.08	0.06, 0.15
SD7	EAS	0.25	0.58	0.08, 0.08	0.09, 0.08	0.03, 0.06	0.10, 0.09	0.07, 0.08	0.01, 0.06	0.08, 0.16
SD8	AFR	0.25	0.54	0.10, 0.09	0.11, 0.10	0.07, 0.07	0.11, 0.09	0.07, 0.07	0.06, 0.07	0.13, 0.19
SD9	AMR	0.50	0.80	0.26, 0.24	0.27, 0.25	0.19, 0.20	0.27, 0.26	0.23, 0.23	0.18, 0.23	0.23, 0.28
SD10	SAS	0.50	0.74	0.25, 0.24	0.26, 0.24	0.17, 0.20	0.28, 0.26	0.20, 0.19	0.15, 0.11	0.18, 0.26
SD11	EAS	0.50	0.42	0.24, 0.23	0.26, 0.25	0.14, 0.12	0.29, 0.27	0.22, 0.15	0.12, 0.07	0.18, 0.26
SD12	AFR	0.50	0.36	0.21, 0.22	0.24, 0.24	0.08, 0.11	0.26, 0.25	0.12, 0.18	0.04, 0.04	0.15, 0.21
SD13	AMR	0.25	0.80	0.11, 0.10	0.11, 0.10	0.07, 0.08	0.11, 0.09	0.06, 0.06	0.07, 0.07	0.08, 0.11
SD14	SAS	0.25	0.74	0.11, 0.09	0.11, 0.09	0.07, 0.08	0.12, 0.10	0.07, 0.06	0.07, 0.08	0.04, 0.12
SD15	EAS	0.25	0.42	0.09, 0.09	0.10, 0.10	0.05, 0.04	0.11, 0.11	0.06, 0.06	0.02, 0.06	0.07, 0.10
SD16	AFR	0.25	0.36	0.08, 0.07	0.09, 0.08	0.05, 0.03	0.10, 0.09	0.07, 0.06	0.04, 0.03	0.02, 0.09

LR: Logistic regression; **DL:** Deep learning; **SD**: Synthetic dataset compendium with a case-to-control ratio of 1:1; **DDP**: Data-disadvantaged population; **AFR**: African; **AMR**: Admixed American; **EAS**: East Asian; **SAS**: South Asian. **Mix0, Mix1, Mix2, Ind1, Ind2, NT, and TL** are the machine learning experiments outlined in Table 2; SD9 and SD13 are same as SD1 and SD5 and are included here for comparison.

Table S13 Multi-ancestral machine learning experiments on synthetic data compendium SD* (Tjur's R^2)

Synthetic Datasets				Tjur's R^2 (LR, DL)						
ID	DDP	h^2	ρ	Mix0	Mix1	Mix2	Ind1	Ind2	NT	TL
SD1*	AMR	0.50	0.80	0.18, 0.24	0.19, 0.25	0.17, 0.20	0.20, 0.26	0.20, 0.26	0.18, 0.22	0.24, 0.28
SD2*	SAS	0.50	0.77	0.20, 0.25	0.20, 0.26	0.17, 0.20	0.20, 0.25	0.25, 0.26	0.14, 0.24	0.23, 0.27
SD3*	EAS	0.50	0.58	0.22, 0.22	0.24, 0.24	0.15, 0.15	0.27, 0.27	0.24, 0.24	0.04, 0.07	0.22, 0.26
SD4*	AFR	0.50	0.54	0.25, 0.23	0.27, 0.24	0.15, 0.17	0.32, 0.24	0.25, 0.17	0.15, 0.21	0.23, 0.29
SD5*	AMR	0.25	0.80	0.06, 0.10	0.07, 0.10	0.02, 0.06	0.07, 0.09	0.05, 0.07	0.01, 0.06	0.04, 0.11
SD6*	SAS	0.25	0.77	0.08, 0.09	0.09, 0.10	0.06, 0.06	0.09, 0.09	0.01, 0.02	0.06, 0.06	0.05, 0.10
SD7*	EAS	0.25	0.58	0.10, 0.08	0.11, 0.10	0.07, 0.06	0.12, 0.10	0.07, 0.09	0.08, 0.06	0.08, 0.12
SD8*	AFR	0.25	0.54	0.05, 0.09	0.05, 0.10	0.04, 0.07	0.06, 0.09	0.04, 0.08	0.02, 0.07	0.08, 0.13
SD9*	AMR	0.50	0.80	0.18, 0.24	0.19, 0.25	0.17, 0.20	0.20, 0.26	0.20, 0.26	0.18, 0.22	0.24, 0.28
SD10*	SAS	0.50	0.74	0.25, 0.24	0.28, 0.24	0.14, 0.20	0.30, 0.26	0.20, 0.19	0.08, 0.11	0.15, 0.26
SD11*	EAS	0.50	0.42	0.15, 0.23	0.16, 0.25	0.11, 0.12	0.17, 0.27	0.19, 0.15	0.10, 0.07	0.19, 0.25
SD12*	AFR	0.50	0.36	0.23, 0.22	0.26, 0.25	0.09, 0.11	0.31, 0.25	0.18, 0.18	0.07, 0.04	0.17, 0.21
SD13*	AMR	0.25	0.80	0.06, 0.10	0.07, 0.10	0.02, 0.06	0.07, 0.09	0.05, 0.07	0.01, 0.06	0.04, 0.11
SD14*	SAS	0.25	0.74	0.06, 0.09	0.05, 0.09	0.05, 0.06	0.05, 0.05	0.02, 0.01	0.08, 0.07	0.06, 0.12
SD15*	EAS	0.25	0.42	0.08, 0.09	0.08, 0.10	0.04, 0.04	0.09, 0.11	0.05, 0.03	0.01, 0.01	0.09, 0.10
SD16*	AFR	0.25	0.36	0.08, 0.07	0.08, 0.08	0.05, 0.05	0.09, 0.09	0.07, 0.05	0.01, 0.02	0.04, 0.07

LR: Logistic regression; **DL:** Deep learning; **SD***: Synthetic dataset compendium with a case-to-control ratio of 1:4; **DDP**: Data-disadvantaged population; **AFR**: African; **AMR**: Admixed American; **EAS**: East Asian; **SAS**: South Asian. **Mix0, Mix1, Mix2, Ind1, Ind2, NT, and TL** are the machine learning experiments outlined in Table 2; SD9* and SD13* are same as SD1* and SD5* and are included here for comparison.

Table S14 Multi-ancestral machine learning experiments on synthetic data compendium SD (PPV)

Synthetic Datasets				Positive Predictive Value (LR, DL)						
**ID**	**DDP**	h^2	ρ	**Mix0**	**Mix1**	**Mix2**	**Ind1**	**Ind2**	**NT**	**TL**
SD1	AMR	0.50	0.80	0.70, 0.71	0.71, 0.72	0.69, 0.69	0.71, 0.71	0.67, 0.67	0.64, 0.65	0.65, 0.73
SD2	SAS	0.50	0.77	0.74, 0.70	0.71, 0.71	0.69, 0.69	0.71, 0.71	0.62, 0.66	0.64, 0.66	0.67, 0.70
SD3	EAS	0.50	0.58	0.68, 0.70	0.69, 0.70	0.65, 0.67	0.72, 0.71	0.67, 0.67	0.64, 0.64	0.63, 0.70
SD4	AFR	0.50	0.54	0.68, 0.70	0.70, 0.71	0.64, 0.68	0.71, 0.71	0.67, 0.62	0.64, 0.54	0.66, 0.69
SD5	AMR	0.25	0.80	0.63, 0.62	0.63, 0.63	0.61, 0.62	0.62, 0.64	0.62, 0.61	0.62, 0.60	0.60, 0.62
SD6	SAS	0.25	0.77	0.60, 0.62	0.62, 0.62	0.60, 0.60	0.63, 0.62	0.61, 0.59	0.59, 0.57	0.58, 0.62
SD7	EAS	0.25	0.58	0.58, 0.60	0.58, 0.60	0.57, 0.58	0.59, 0.60	0.57, 0.58	0.54, 0.40	0.55, 0.61
SD8	AFR	0.25	0.54	0.63, 0.61	0.64, 0.63	0.61, 0.61	0.63, 0.62	0.61, 0.55	0.61, 0.61	0.61, 0.63
SD9	AMR	0.50	0.80	0.70, 0.71	0.71, 0.72	0.69, 0.69	0.71, 0.71	0.67, 0.67	0.64, 0.65	0.65, 0.73
SD10	SAS	0.50	0.74	0.71, 0.71	0.72, 0.71	0.67, 0.68	0.72, 0.71	0.65, 0.68	0.64, 0.66	0.66, 0.71
SD11	EAS	0.50	0.42	0.70, 0.69	0.71, 0.71	0.62, 0.64	0.70, 0.71	0.65, 0.66	0.60, 0.59	0.62, 0.67
SD12	AFR	0.50	0.36	0.68, 0.68	0.70, 0.70	0.58, 0.63	0.72, 0.71	0.67, 0.66	0.57, 0.58	0.60, 0.70
SD13	AMR	0.25	0.80	0.63, 0.62	0.63, 0.63	0.61, 0.62	0.62, 0.64	0.62, 0.61	0.62, 0.60	0.60, 0.62
SD14	SAS	0.25	0.74	0.61, 0.63	0.64, 0.65	0.60, 0.61	0.65, 0.66	0.60, 0.61	0.61, 0.60	0.62, 0.65
SD15	EAS	0.25	0.42	0.61, 0.60	0.63, 0.64	0.60, 0.61	0.63, 0.63	0.60, 0.60	0.55, 0.55	0.59, 0.62
SD16	AFR	0.25	0.36	0.61, 0.60	0.62, 0.60	0.57, 0.57	0.59, 0.60	0.54, 0.55	0.58, 0.31	0.60, 0.59

LR: Logistic regression; **DL:** Deep learning; **SD**: Synthetic dataset compendium with a case-to-control ratio of 1:1; **DDP**: Data-disadvantaged population; **AFR**: African; **AMR**: Admixed American; **EAS**: East Asian; **SAS**: South Asian. **Mix0, Mix1, Mix2, Ind1, Ind2, NT, and TL** are the machine learning experiments outlined in Table 2; SD9 and SD13 are same as SD1 and SD5 and are included here for comparison.

Table S15 Multi-ancestral machine learning experiments on synthetic data compendium SD* (PPV)

Synthetic Datasets				Positive Predictive Value (LR, DL)						
ID	DDP	h^2	ρ	Mix0	Mix1	Mix2	Ind1	Ind2	NT	TL
SD1*	AMR	0.50	0.80	0.34, 0.35	0.35, 0.35	0.26, 0.25	0.35, 0.36	0.23, 0.27	0.24, 0.26	0.28, 0.38
SD2*	SAS	0.50	0.77	0.35, 0.38	0.35, 0.38	0.27, 0.28	0.36, 0.37	0.27, 0.26	0.25, 0.26	0.33, 0.38
SD3*	EAS	0.50	0.58	0.36, 0.40	0.37, 0.40	0.30, 0.30	0.40, 0.40	0.29, 0.28	0.30, 0.31	0.30, 0.41
SD4*	AFR	0.50	0.54	0.41, 0.41	0.42, 0.42	0.34, 0.33	0.41, 0.41	0.33, 0.34	0.32, 0.20	0.34, 0.39
SD5*	AMR	0.25	0.80	0.27, 0.28	0.29, 0.29	0.23, 0.22	0.26, 0.29	0.25, 0.24	0.23, 0.24	0.23, 0.27
SD6*	SAS	0.25	0.77	0.28, 0.29	0.28, 0.29	0.21, 0.20	0.27, 0.29	0.23, 0.21	0.20, 0.20	0.21, 0.28
SD7*	EAS	0.25	0.58	0.31, 0.33	0.31, 0.33	0.21, 0.25	0.31, 0.33	0.22, 0.24	0.28, 0.24	0.27, 0.32
SD8*	AFR	0.25	0.54	0.27, 0.26	0.29, 0.33	0.20, 0.20	0.27, 0.31	0.23, 0.24	0.23, 0.24	0.25, 0.30
SD9*	AMR	0.50	0.80	0.34, 0.35	0.35, 0.35	0.26, 0.25	0.35, 0.36	0.23, 0.27	0.24, 0.26	0.28, 0.38
SD10*	SAS	0.50	0.74	0.40, 0.38	0.42, 0.39	0.34, 0.34	0.40, 0.43	0.31, 0.31	0.31, 0.33	0.30, 0.37
SD11*	EAS	0.50	0.42	0.35, 0.35	0.35, 0.36	0.30, 0.31	0.34, 0.36	0.28, 0.29	0.28, 0.30	0.31, 0.36
SD12*	AFR	0.50	0.36	0.37, 0.38	0.40, 0.40	0.28, 0.31	0.41, 0.40	0.33, 0.31	0.26, 0.28	0.25, 0.37
SD13*	AMR	0.25	0.80	0.27, 0.28	0.29, 0.29	0.23, 0.22	0.26, 0.29	0.25, 0.24	0.23, 0.24	0.23, 0.27
SD14*	SAS	0.25	0.74	0.27, 0.27	0.30, 0.31	0.25, 0.25	0.30, 0.31	0.27, 0.26	0.27, 0.27	0.29, 0.32
SD15*	EAS	0.25	0.42	0.29, 0.29	0.29, 0.31	0.22, 0.22	0.28, 0.30	0.21, 0.23	0.21, 0.22	0.24, 0.28
SD16*	AFR	0.25	0.36	0.29, 0.30	0.30, 0.32	0.21, 0.23	0.30, 0.32	0.21, 0.21	0.22, 0.24	0.24, 0.30

LR: Logistic regression; **DL:** Deep learning; **SD***: Synthetic dataset compendium with a case-to-control ratio of 1:4; **DDP**: Data-disadvantaged population; **AFR**: African; **AMR**: Admixed American; **EAS**: East Asian; **SAS**: South Asian. **Mix0, Mix1, Mix2, Ind1, Ind2, NT, and TL** are the machine learning experiments outlined in Table 2; SD9* and SD13* are same as SD1* and SD5* and are included here for comparison.

Table S16 Multi-ancestral machine learning experiments on synthetic data compendium SD (NPV)

Synthetic Datasets				Negative Predictive Value (LR, DL)						
**ID**	**DDP**	h^2	ρ	**Mix0**	**Mix1**	**Mix2**	**Ind1**	**Ind2**	**NT**	**TL**
SD1	AMR	0.50	0.80	0.69, 0.73	0.70, 0.73	0.69, 0.67	0.71, 0.72	0.69, 0.70	0.63, 0.65	0.58, 0.72
SD2	SAS	0.50	0.77	0.76, 0.69	0.69, 0.71	0.65, 0.67	0.71, 0.72	0.64, 0.64	0.66, 0.62	0.66, 0.69
SD3	EAS	0.50	0.58	0.67, 0.72	0.70, 0.72	0.64, 0.64	0.75, 0.71	0.66, 0.66	0.64, 0.68	0.58, 0.69
SD4	AFR	0.50	0.54	0.67, 0.71	0.70, 0.72	0.60, 0.67	0.71, 0.71	0.65, 0.66	0.62, 0.52	0.61, 0.67
SD5	AMR	0.25	0.80	0.66, 0.64	0.67, 0.67	0.56, 0.62	0.66, 0.66	0.65, 0.65	0.61, 0.55	0.64, 0.65
SD6	SAS	0.25	0.77	0.57, 0.64	0.61, 0.64	0.56, 0.62	0.65, 0.65	0.65, 0.54	0.57, 0.58	0.51, 0.63
SD7	EAS	0.25	0.58	0.58, 0.63	0.59, 0.58	0.53, 0.55	0.54, 0.59	0.55, 0.54	0.51, 0.47	0.52, 0.59
SD8	AFR	0.25	0.54	0.67, 0.69	0.68, 0.70	0.62, 0.62	0.67, 0.69	0.67, 0.67	0.67, 0.62	0.67, 0.68
SD9	AMR	0.50	0.80	0.69, 0.73	0.70, 0.73	0.69, 0.67	0.71, 0.72	0.69, 0.70	0.63, 0.65	0.58, 0.72
SD10	SAS	0.50	0.74	0.73, 0.71	0.72, 0.72	0.68, 0.69	0.73, 0.73	0.66, 0.69	0.65, 0.66	0.67, 0.72
SD11	EAS	0.50	0.42	0.70, 0.70	0.70, 0.70	0.58, 0.61	0.70, 0.70	0.60, 0.65	0.55, 0.59	0.62, 0.67
SD12	AFR	0.50	0.36	0.69, 0.69	0.71, 0.69	0.54, 0.58	0.72, 0.71	0.68, 0.67	0.63, 0.58	0.58, 0.71
SD13	AMR	0.25	0.80	0.66, 0.64	0.67, 0.67	0.56, 0.62	0.66, 0.66	0.65, 0.65	0.61, 0.55	0.64, 0.65
SD14	SAS	0.25	0.74	0.67, 0.67	0.65, 0.66	0.63, 0.61	0.68, 0.66	0.62, 0.59	0.64, 0.63	0.60, 0.64
SD15	EAS	0.25	0.42	0.63, 0.60	0.65, 0.66	0.55, 0.62	0.67, 0.66	0.57, 0.60	0.53, 0.56	0.59, 0.63
SD16	AFR	0.25	0.36	0.62, 0.62	0.64, 0.64	0.60, 0.61	0.62, 0.63	0.61, 0.57	0.57, 0.61	0.59, 0.61

LR: Logistic regression; **DL:** Deep learning; **SD**: Synthetic dataset compendium with a case-to-control ratio of 1:1; **DDP**: Data-disadvantaged population; **AFR**: African; **AMR**: Admixed American; **EAS**: East Asian; **SAS**: South Asian. **Mix0, Mix1, Mix2, Ind1, Ind2, NT, and TL** are the machine learning experiments outlined in Table 2; SD9 and SD13 are same as SD1 and SD5 and are included here for comparison.

Table S17 Multi-ancestral machine learning experiments on synthetic data compendium SD* (NPV)

Synthetic Datasets				Negative Predictive Value (LR, DL)						
**ID**	**DDP**	h^2	ρ	**Mix0**	**Mix1**	**Mix2**	**Ind1**	**Ind2**	**NT**	**TL**
SD1*	AMR	0.50	0.80	0.67, 0.71	0.65, 0.72	0.68, 0.69	0.66, 0.72	0.63, 0.65	0.65, 0.70	0.69, 0.72
SD2*	SAS	0.50	0.77	0.71, 0.69	0.72, 0.70	0.66, 0.68	0.70, 0.70	0.68, 0.64	0.65, 0.63	0.56, 0.69
SD3*	EAS	0.50	0.58	0.65, 0.71	0.67, 0.70	0.52, 0.65	0.71, 0.72	0.64, 0.60	0.60, 0.61	0.56, 0.68
SD4*	AFR	0.50	0.54	0.71, 0.72	0.71, 0.71	0.68, 0.66	0.71, 0.73	0.60, 0.62	0.66, 0.55	0.66, 0.69
SD5*	AMR	0.25	0.80	0.60, 0.65	0.61, 0.65	0.55, 0.63	0.59, 0.65	0.57, 0.63	0.57, 0.57	0.59, 0.64
SD6*	SAS	0.25	0.77	0.59, 0.62	0.59, 0.61	0.58, 0.59	0.53, 0.60	0.56, 0.56	0.57, 0.58	0.56, 0.59
SD7*	EAS	0.25	0.58	0.64, 0.61	0.65, 0.66	0.56, 0.58	0.64, 0.64	0.63, 0.63	0.57, 0.60	0.59, 0.65
SD8*	AFR	0.25	0.54	0.62, 0.61	0.59, 0.61	0.57, 0.56	0.59, 0.63	0.56, 0.59	0.59, 0.56	0.57, 0.63
SD9*	AMR	0.50	0.80	0.67, 0.71	0.65, 0.72	0.68, 0.69	0.66, 0.72	0.63, 0.65	0.65, 0.70	0.69, 0.72
SD10*	SAS	0.50	0.74	0.70, 0.70	0.73, 0.71	0.63, 0.69	0.75, 0.72	0.64, 0.68	0.57, 0.66	0.68, 0.70
SD11*	EAS	0.50	0.42	0.70, 0.70	0.70, 0.72	0.63, 0.61	0.70, 0.73	0.66, 0.65	0.54, 0.59	0.64, 0.67
SD12*	AFR	0.50	0.36	0.66, 0.67	0.70, 0.68	0.63, 0.61	0.71, 0.70	0.67, 0.67	0.56, 0.56	0.55, 0.68
SD13*	AMR	0.25	0.80	0.60, 0.65	0.61, 0.65	0.55, 0.63	0.59, 0.65	0.57, 0.63	0.57, 0.57	0.59, 0.64
SD14*	SAS	0.25	0.74	0.61, 0.63	0.63, 0.64	0.55, 0.56	0.63, 0.62	0.61, 0.59	0.62, 0.59	0.55, 0.62
SD15*	EAS	0.25	0.42	0.60, 0.61	0.59, 0.62	0.60, 0.57	0.59, 0.62	0.56, 0.58	0.54, 0.54	0.53, 0.61
SD16*	AFR	0.25	0.36	0.62, 0.60	0.60, 0.58	0.63, 0.58	0.60, 0.60	0.57, 0.55	0.56, 0.53	0.56, 0.60

LR: Logistic regression; **DL:** Deep learning; **SD***: Synthetic dataset compendium with a case-to-control ratio of 1:4; **DDP**: Data-disadvantaged population; **AFR**: African; **AMR**: Admixed American; **EAS**: East Asian; **SAS**: South Asian. **Mix0, Mix1, Mix2, Ind1, Ind2, NT, and TL** are the machine learning experiments outlined in Table 2; SD9* and SD13* are same as SD1* and SD5* and are included here for comparison.

Table S18 Disparity detection and mitigation in multi-ancestral machine learning using synthetic datasets

Key observations	Comparison	Mean Difference in AUPR		Mean Difference in Tjur's R^2		Mean Difference in PPV		Mean Difference in NPV	
		**SD**	**SD***	**SD**	**SD***	**SD**	**SD**	**SD***	**SD***
Performance disparity gap between EUR and DDPs in mixture and independent learning	Mix1_LR vs Mix2_LR	**0.08**	**0.07**	**0.07**	**0.06**	**0.04**	**0.08**	**0.07**	**0.04**
	Mix1_DL vs Mix2_DL	**0.05**	**0.05**	**0.05**	**0.06**	**0.03**	**0.08**	**0.05**	**0.05**
	Ind1_LR vs Ind2_LR	**0.07**	**0.09**	**0.06**	**0.03**	**0.04**	**0.07**	**0.04**	**0.04**
	Ind1_DL vs Ind2_DL	**0.07**	**0.07**	**0.04**	**0.04**	**0.04**	**0.08**	**0.05**	**0.05**
Improvement from DL-based transfer learning	TL_DL vs Mix2_DL	**0.02**	**0.04**	**0.08**	**0.07**	**0.03**	**0.07**	**0.04**	**0.04**
	TL_DL vs Ind2_DL	**0.04**	**0.06**	**0.06**	**0.05**	**0.03**	**0.07**	**0.03**	**0.04**
	TL_DL vs NT_DL	**0.09**	**0.10**	**0.08**	**0.09**	**0.08**	**0.08**	**0.07**	**0.06**
Improvement from LR-based transfer learning	TL_LR vs Mix2_LR	-0.01	-0.03	**0.02**	**0.04**	-0.01	**0.02**	0.00	-0.01
	TL_LR vs Ind2_LR	-0.01	-0.02	0.00	0.01	-0.01	0.01	-0.04	-0.01
	TL_LR vs NT_LR	**0.02**	0.00	**0.04**	**0.06**	**0.01**	**0.02**	0.00	0.01
Performance difference between DL- and LR-based transfer learning	TL_DL vs TL_LR	**0.05**	**0.10**	**0.06**	**0.05**	**0.04**	**0.06**	**0.06**	**0.06**

The statistically significant (p-value less than 0.05) performance differences are highlighted using bold font. **LR:** Logistic regression; **DL:** Deep learning; **SD**: Synthetic dataset compendium with a case-to-control ratio of 1:1; **SD***: Synthetic dataset compendium with a case-to-control ratio of 1:4; **Mix0, Mix1, Mix2, Ind1, Ind2, NT, and TL** are the machine learning experiments outlined in Table 2.

Table S19 Comparison of the performance of DL- and LR-based models across synthetic datasets

Learning scheme	Comparison	Mean Difference in AUROC		Mean Difference in AUPR		Mean Difference in Tjur's R^2		Mean Difference in PPV		Mean Difference in NPV	
		SD	SD*	SD	SD*	SD	SD*	SD	SD*	SD	SD*
Mixture learning	Mix0_DL vs Mix0_LR	**0.01**	**0.01**	0.00	**0.01**	-0.01	**0.02**	0.00	**0.01**	0.01	**0.02**
	Mix1_DL vs Mix1_LR	**0.01**	**0.01**	-0.01	0.01	-0.01	**0.02**	0.00	**0.01**	**0.01**	**0.02**
	Mix2_DL vs Mix2_LR	**0.01**	**0.02**	**0.02**	**0.02**	0.00	**0.02**	**0.01**	0.00	**0.03**	0.02
Independent learning	Ind1_DL vs Ind1_LR	**0.01**	**0.01**	-0.01	-0.01	-0.02	0.01	0.00	**0.02**	0.00	**0.02**
	Ind2_DL vs Ind2_LR	0.00	0.01	-0.01	0.01	-0.00	0.00	0.00	0.00	0.00	0.01
Naïve transfer	NT_DL vs NT_LR	-0.01	0.00	-0.02	-0.01	0.01	**0.02**	-0.03	0.00	-0.01	0.01
Transfer learning	TL_DL vs TL_LR	**0.05**	**0.07**	**0.05**	**0.10**	**0.06**	**0.05**	**0.04**	**0.06**	**0.06**	**0.06**

The statistically significant (p-value less than 0.05) performance differences are highlighted using bold font. **LR:** Logistic regression; **DL:** Deep learning; **SD**: Synthetic dataset compendium with a case-to-control ratio of 1:1; **SD***: Synthetic dataset compendium with a case-to-control ratio of 1:4; **Mix0, Mix1, Mix2, Ind1, Ind2, NT, and TL** are the machine learning experiments outlined in Table 2.

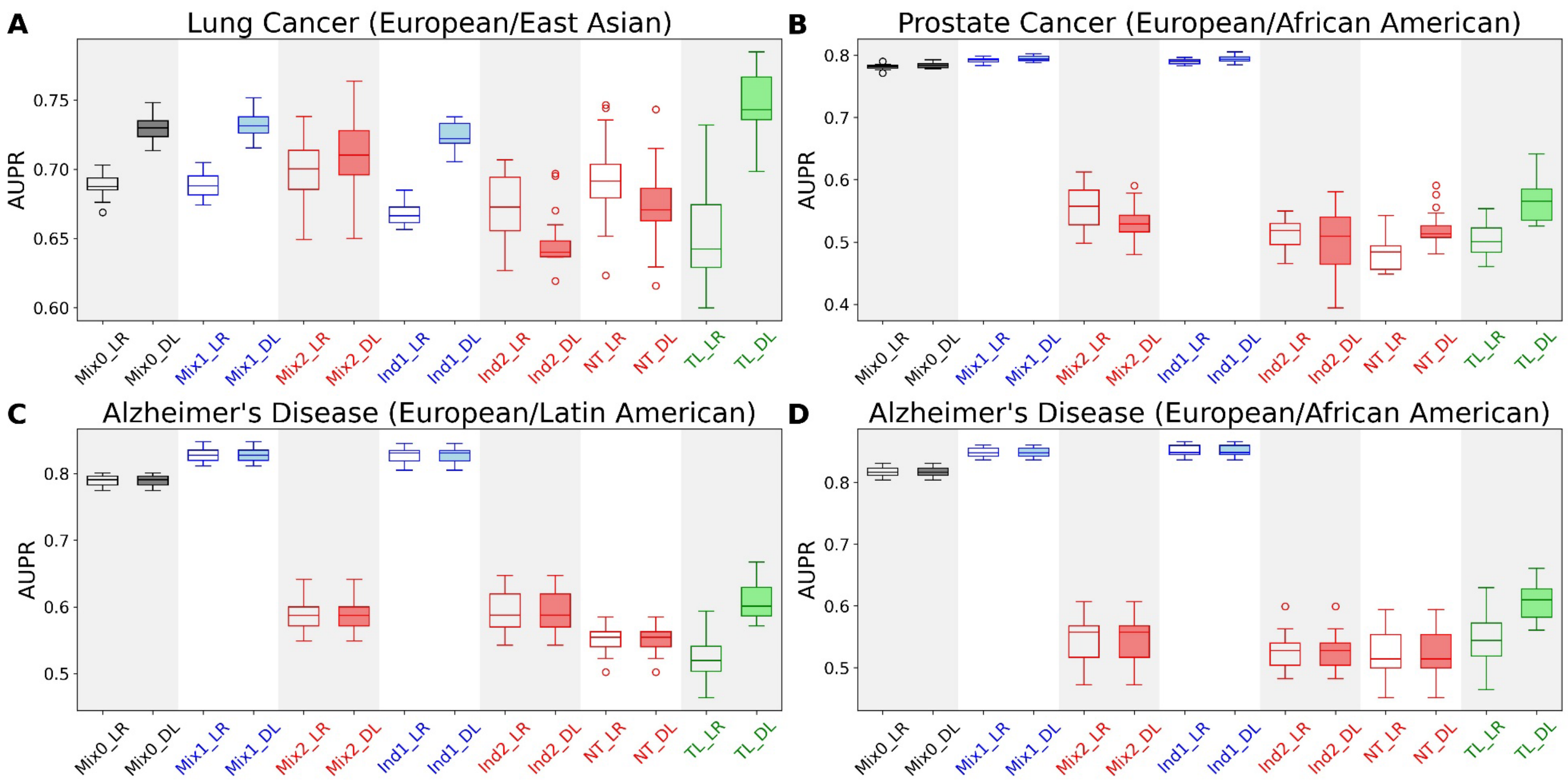

Fig. S1 Multi-ancestral clinico-genomic prediction of **(A)** Lung Cancer involving European and East Asian populations, **(B)** Prostate Cancer involving European and African American populations, **(C)** Alzheimer's Disease involving European and Latin American populations, and **(D)** Alzheimer's Disease involving European and African American populations. Each box plot represents the machine learning model performance (AUPR) of 20 independent runs. **LR:** Logistic regression; **DL:** Deep learning. **Mix0, Mix1, Mix2, Ind1, Ind2, NT, and TL** are the machine learning experiments outlined in Table 2.

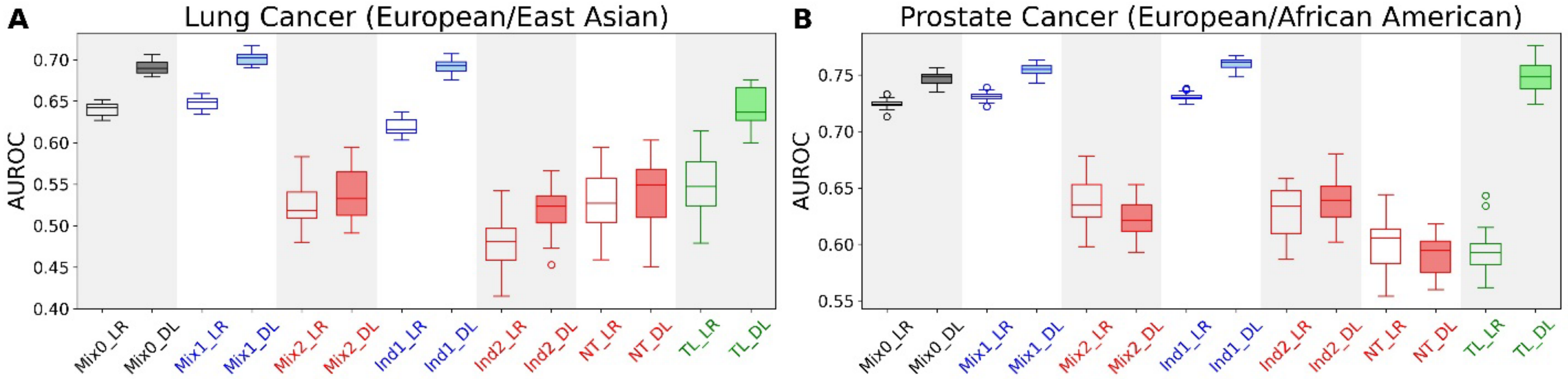

Fig. S2 Multi-ancestral clinico-genomic prediction of **(A)** Lung Cancer involving European and East Asian populations (1000 SNPs), **(B)** Prostate Cancer involving European and African American populations (1000 SNPs). Each box plot represents the machine learning model performance (AUROC) of 20 independent runs. **LR:** Logistic regression; **DL:** Deep learning. **Mix0, Mix1, Mix2, Ind1, Ind2, NT, and TL** are the machine learning experiments outlined in Table 2.

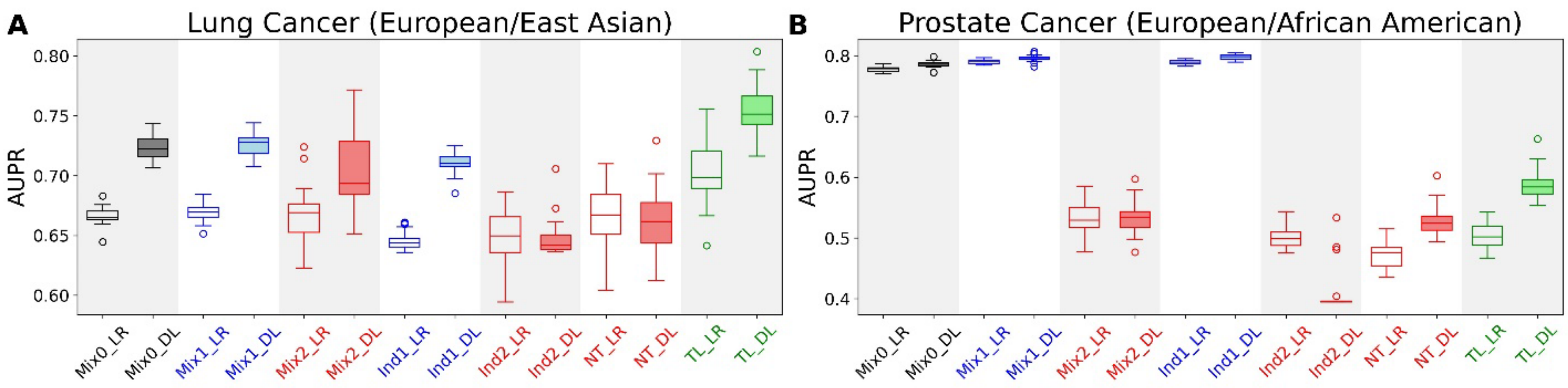

Fig. S3 Multi-ancestral clinico-genomic prediction of **(A)** Lung Cancer involving European and East Asian populations (1000 SNPs), **(B)** Prostate Cancer involving European and African American populations (1000 SNPs). Each box plot represents the machine learning model performance (AUPR) of 20 independent runs. **LR:** Logistic regression; **DL:** Deep learning. **Mix0, Mix1, Mix2, Ind1, Ind2, NT, and TL** are the machine learning experiments outlined in Table 2.

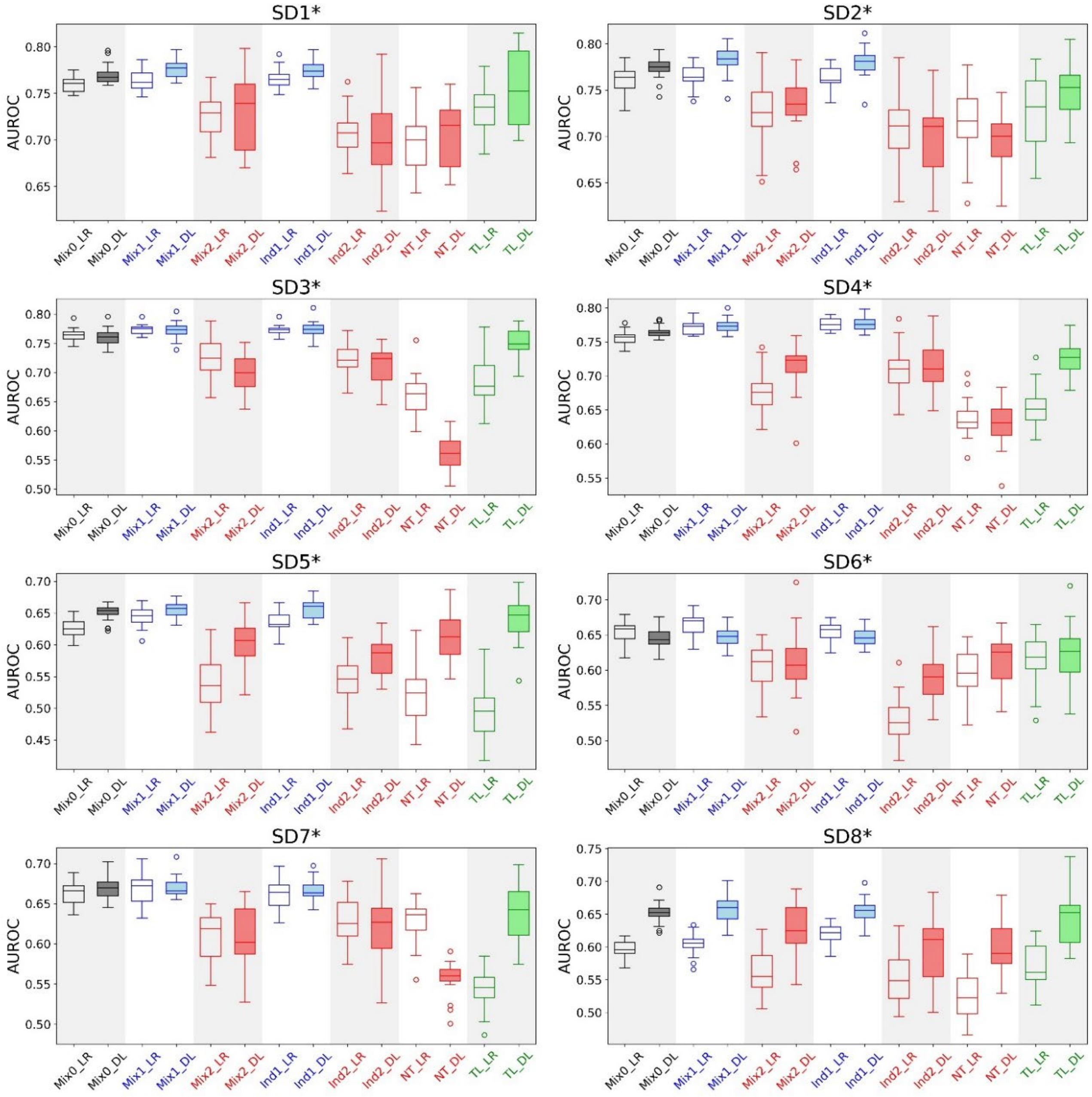

The figure continues on the next page.

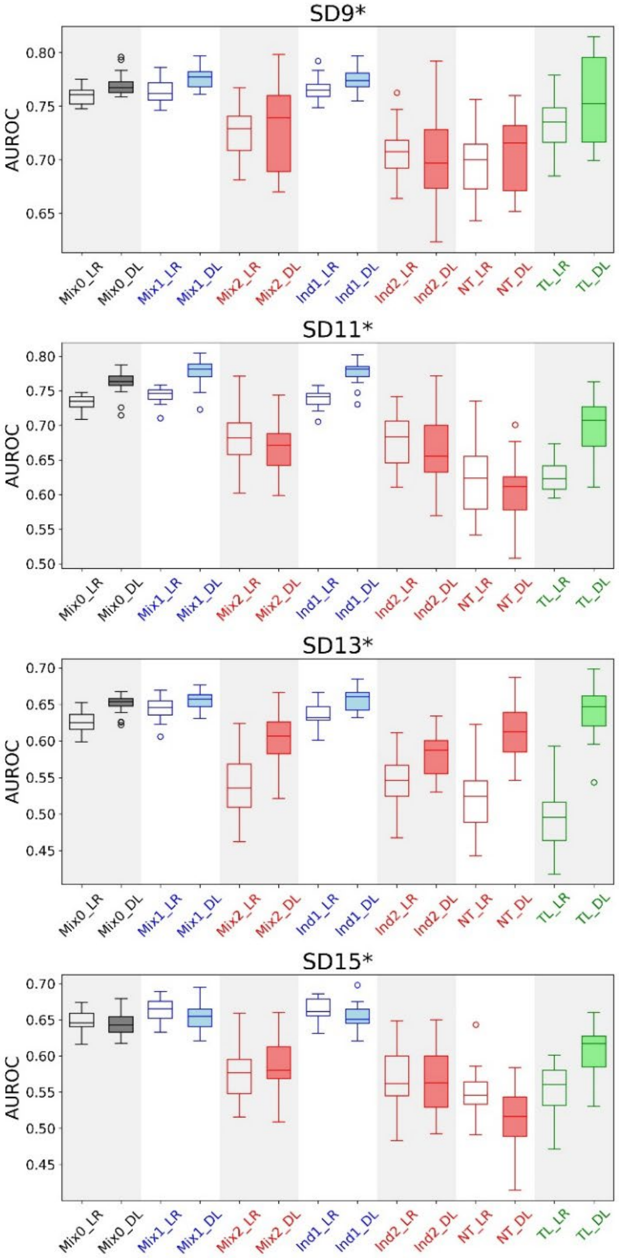

Fig. S4 Multi-ancestral machine learning experiments on synthetic dataset compendium SD*. Each box plot represents the machine learning model performance (AUROC) of 20 independent runs. **LR:** Logistic regression; **DL:** Deep learning. **Mix0, Mix1, Mix2, Ind1, Ind2, NT, and TL** are the machine learning experiments outlined in Table 2.

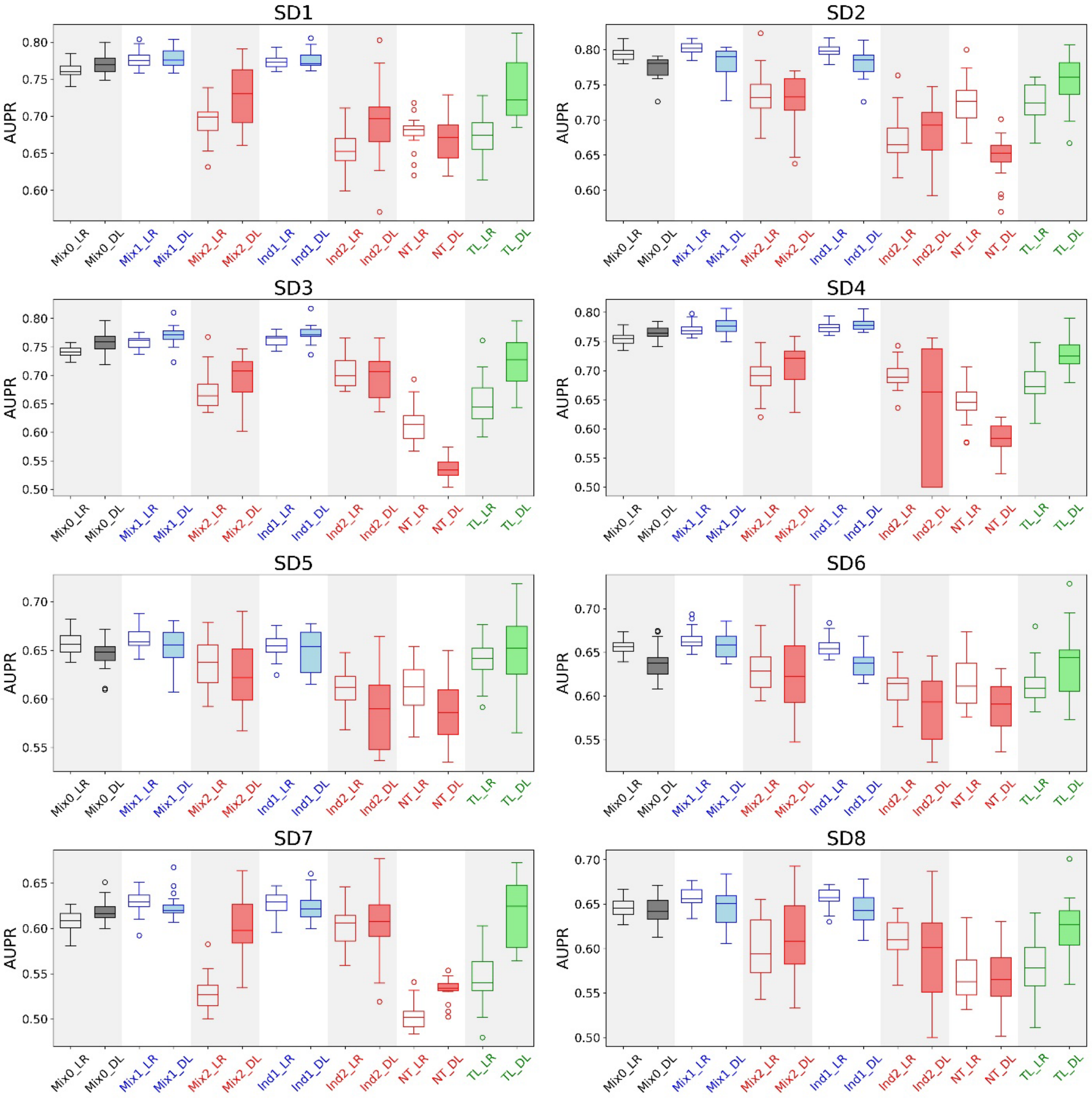

The figure continues on the next page.

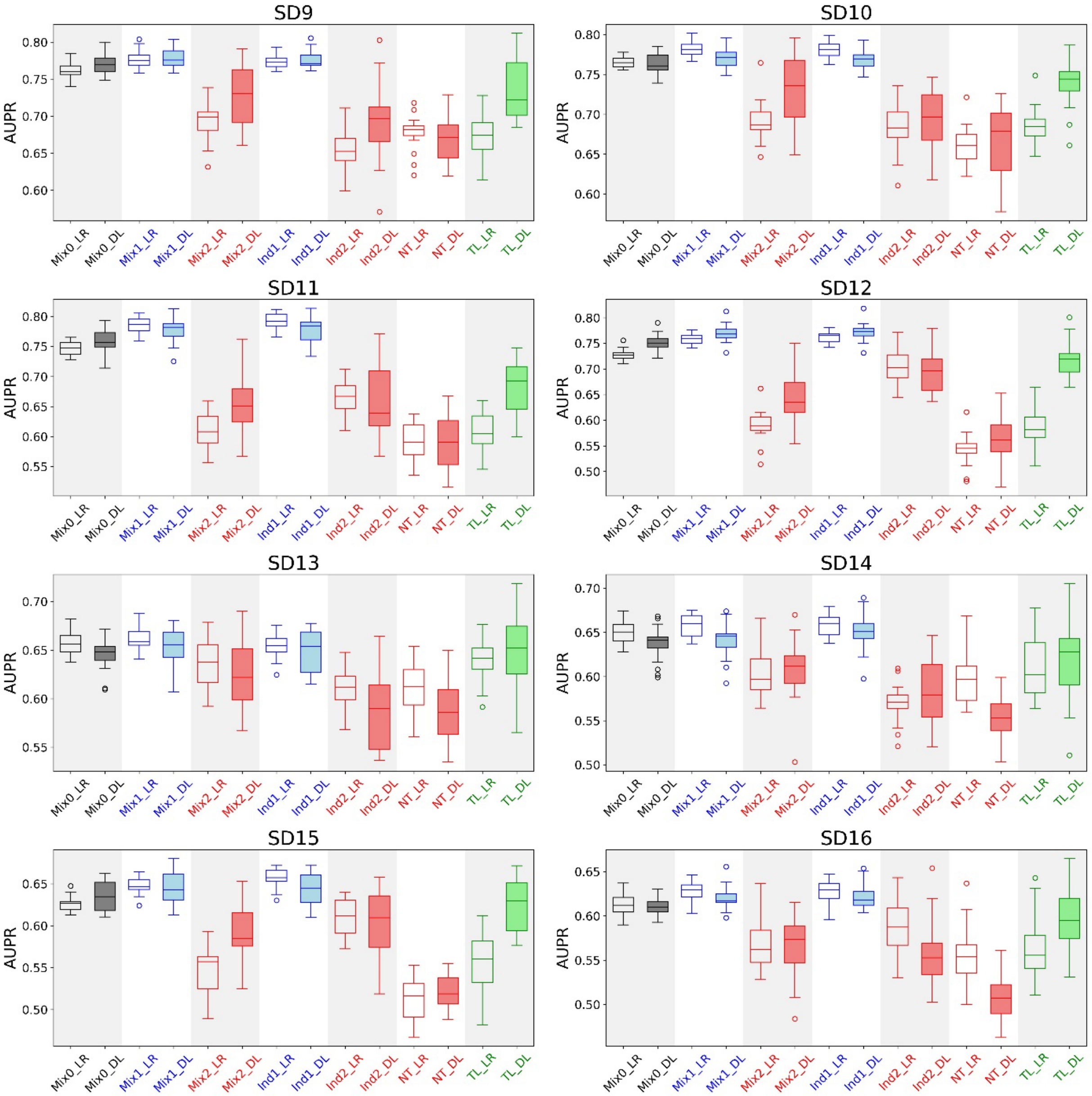

Fig. S5 Multi-ancestral machine learning experiments on the synthetic dataset compendium SD. Each box plot represents the machine learning model performance (AUPR) of 20 independent runs. **LR:** Logistic regression; **DL:** Deep learning. **Mix0, Mix1, Mix2, Ind1, Ind2, NT, and TL** are the machine learning experiments outlined in Table 2.

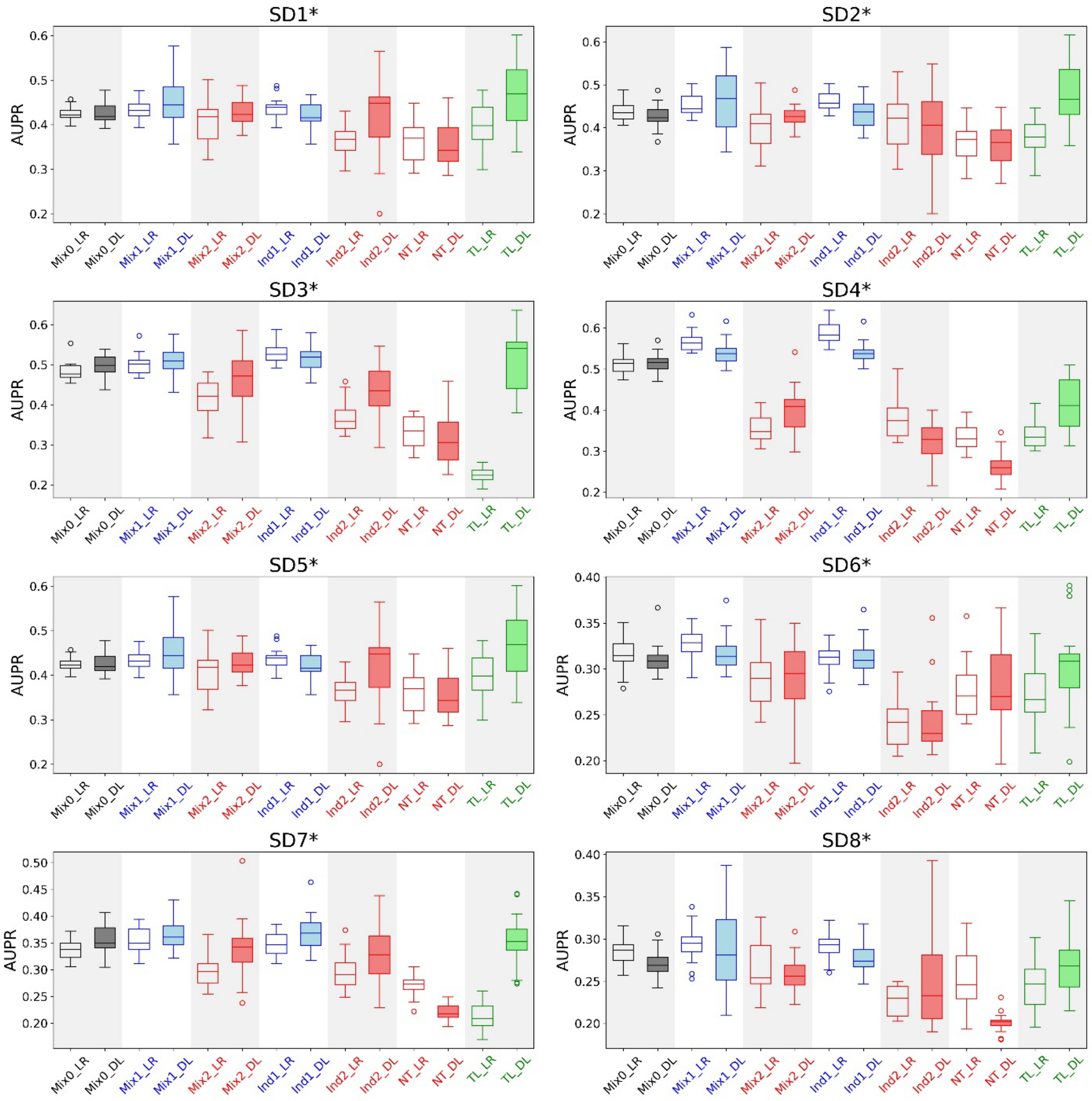

The figure continues on the next page.

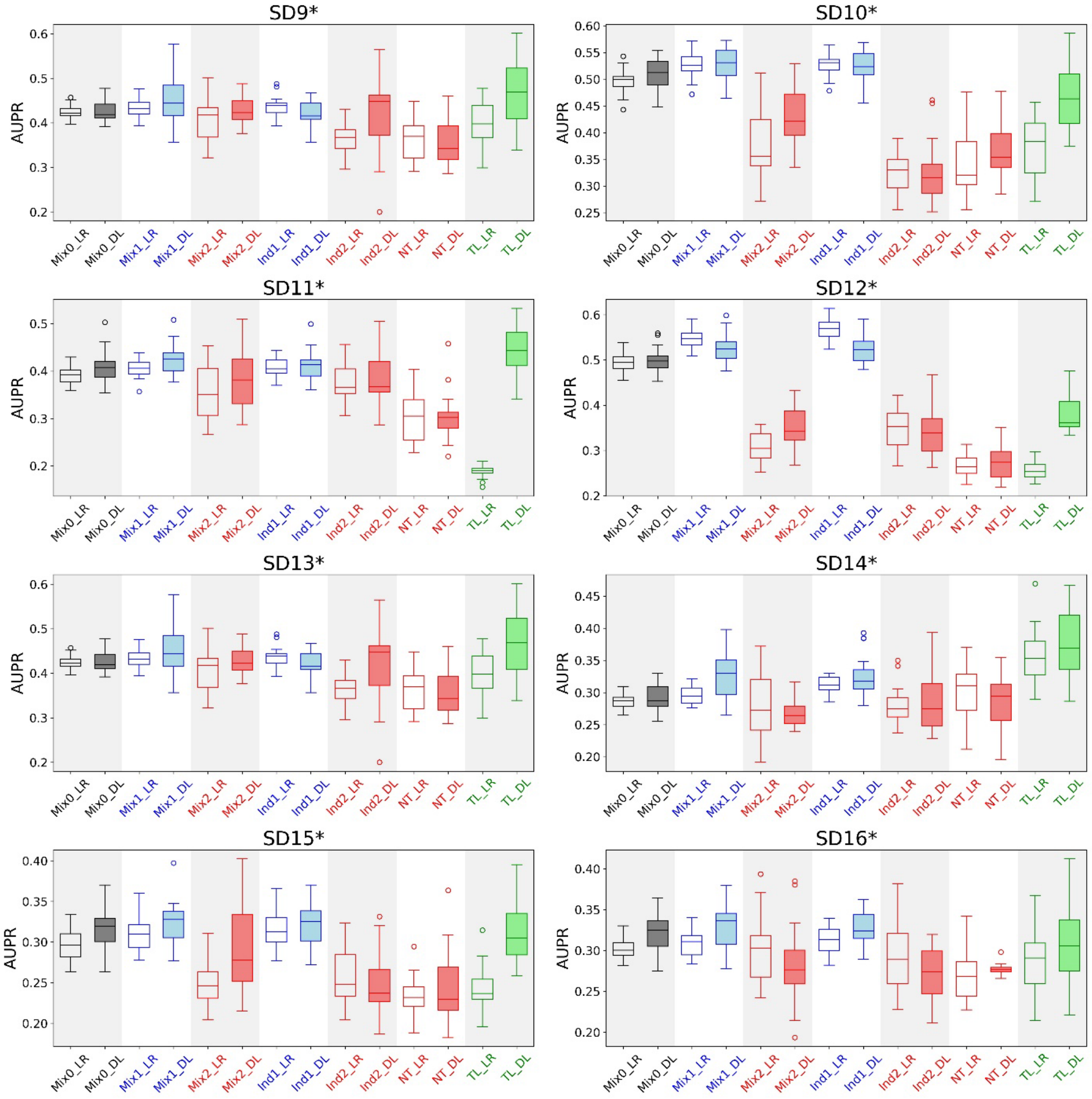

Fig. S6 Multi-ancestral machine learning experiments on the synthetic dataset compendium SD*. Each box plot represents the machine learning model performance (AUPR) of 20 independent runs. **LR:** Logistic regression; **DL:** Deep learning. **Mix0, Mix1, Mix2, Ind1, Ind2, NT, and TL** are the machine learning experiments outlined in Table 2.
